# Inhibiting microtubule polymerization with EAPB02303, a prodrug activated by catechol-O-methyl transferase, enhances paclitaxel effect in pancreatic cancer models

**DOI:** 10.1038/s41419-025-07747-1

**Published:** 2025-06-09

**Authors:** Kévin Bigot, Cindy Patinote, Véronique Garambois, Adrien Chouchou, Stéphanie Gayraud-Paniagua, Nadia Vie, Yann Maggipinto, Elias Smyej, Mathilde Robin, Margot Machu, Marine Bruciamacchie, Pierre-Emmanuel Colombo, Corinne Bousquet, Muriel Mathonnet, Ela Levy-Augé, Diego Tosi, Pierre-Antoine Bonnet, Céline Gongora, Carine Deleuze-Masquéfa, Christel Larbouret

**Affiliations:** 1 IRCM, Université de Montpellier, Inserm, ICM, Montpellier, France; 2 Institut des Biomolécules Max Mousseron (IBMM), UMR 5247, (CNRS, ENSCM, Université de Montpellier), Montpellier, France; 3 Université Toulouse III-Paul Sabatier - Centre de Recherche en Cancérologie de Toulouse (CRCT) – UMR1037 Inserm– UMR, 5071 CNRS Toulouse, France; 4 INSERM UMLR-1308, University of Limoges, Limoges, France; 5 IRCM, Université de Montpellier, Inserm, ICM, CNRS, Montpellier, France

**Keywords:** Mechanism of action, Drug development

## Abstract

The Imiqualines family is an original group of small heterocyclic compounds, diversely substituted around different scaffolds. Among these compounds, the lead EAPB02303 displays outstanding cytotoxic activity at nanomolar concentrations comparable to those of standard-of-care chemotherapy drugs in different cancer cell lines, including Pancreatic Ductal AdenoCarcinoma (PDAC) cell lines. Due to its high aggressiveness and resistance to therapies, PDAC has an extremely poor prognosis with limited treatment options. Here, we demonstrated the cytotoxic activities of EAPB02303 alone or combined with standard chemotherapy drugs in several PDAC cell lines and confirmed these results in patient-derived xenograft mouse models. EAPB02303 potently induced cell cycle arrest in the G2/M phase and in mitosis followed by apoptosis. Then, using a combination of transcriptomic, proteomic, biochemical and cellular assay, we found that EAPB02303 mechanism of action relies on its bioactivation by catechol-O-methyltransferase, resulting in the production of a methylated compound that effectively inhibits microtubule polymerization. Moreover, EAPB02303 had a synergistic effect when combined with paclitaxel (the standard-of-care agent in PDAC) providing the rationale to continue the development of EAPB02303 combination strategies for the treatment of catechol-O-methyltransferase-overexpressing PDAC.

## Introduction

The Imiqualine family includes heterocyclic molecules based on scaffolds diversely substituted, and divided into five series: imidazo[1,2-*a*]quinoxaline, imidazo[1,2-*a*]pyrazine, imidazo[1,5-*a*]quinoxaline, [1,2,4]triazolo[4,3-*a*]quinoxaline and pyrazolo[1,5-*a*]quinoxaline. Among these compounds, first-generation hits, such as EAPB0503 and EAPB0203 belonging to the imidazo[1,2-*a*]quinoxaline series, showed cytotoxic activities at submicromolar concentrations in different cancer cell lines that were higher than those of reference molecules (vemurafenib, fotemustine, dacarbazine) in melanoma cell lines [1, 2]. These compounds inhibit microtubule polymerization by binding to the colchicine binding site in β-tubulin [3]. Additional drug design studies led to the development of second-generation Imiqualine compounds with significantly higher cytotoxic activities. The most potent molecule, EAPB02303, demonstrates remarkable activity at nanomolar concentrations in different cancer cell lines, including Pancreatic Ductal Adenocarcinoma (PDAC) cell lines. In vivo, EAPB02303 reduced tumor size in mice xenografted with A375 human melanoma cells in a dose-dependent manner. Moreover, its terminal half-life following intraperitoneal administration in mice was 6 h using a dosage method validation according to the FDA and EMA guidelines [4, 5]. Comparison of the transcriptomic profiles induced by EAPB02303 and twelve well-known anti-cancer agents (*e.g*. microtubule disruptors, alkylating agents, anti-metabolic agents, targeted therapies) was performed and highlighted significant differences between EAPB02303 and these known anti-cancer agents. Furthermore, EAPB02303 failed to inhibit microtubule polymerization when added to purified tubulin using a standard polymerization protocol [4]. Therefore, the mechanism of action underpinning EAPB02303 cytotoxic activity must be elucidated for its optimal pre-clinical development.

By 2030, pancreatic cancer could rise to second place among the causes of cancer mortality, making it one of the major public health problems [6]. PDAC has an extremely poor prognosis, because of late diagnosis and broad primary resistance to therapies [7]. Surgery is the only potentially curative treatment when PDAC is diagnosed at very early stage of development. The current standard of care includes gemcitabine alone or combined with the nanoparticle albumin-bound form of the microtubule-stabilizing agent paclitaxel (*i.e. nab-*paclitaxel) and FOLFIRINOX (5-fluorouracil, leucovorin, irinotecan and oxaliplatin) [8]. In all cases, these chemotherapy combinations increase patient survival, but the response is relatively poor with significant toxicity [9]. Furthermore, acquired and innate resistance are usual in PDAC, and the development of new active compounds remains a challenge that requires extensive research.

Due to its high activity in PDAC cell lines, EAPB02303 is an interesting candidate for pre-clinical studies. Here, we thoroughly evaluated EAPB02303 effects in PDAC cell lines and mouse models and investigated its mechanism of action. First, we measured the cytotoxic activity of EAPB02303 alone or combined with standard chemotherapy drugs in 2D and 3D (spheroid) cultures of PDAC cell lines. Then, we evaluated its effect in patient-derived xenograft (PDX) mouse models. We showed a potent cytotoxic activity of EAPB02303 in combination with paclitaxel in vitro and in mice. Using a combination of transcriptomic, proteomic, biochemical and cellular assay, we demonstrated that EAPB02303 mechanism of action relies on its bioactivation by catechol-O-methyltransferase (COMT), resulting in the production of a methylated compound that effectively inhibits microtubule polymerization. Analysis of The Cancer Genome Atlas (TCGA) data indicated that COMT is upregulated in PDAC compared with healthy tissue samples and this is associated with poor prognosis.

## Materials and methods

Cell lines, drug reagents, 2D and 3D cell growth inhibition assay, flow cytometry, whole-cell microtubule analysis, western blotting, Cellular thermal shift assay (CETSA), RNA sequencing (RNA-seq) and data analysis, Reverse-phase protein array (RPPA), CRISPR-Cas9-mediated knock-out of *COMT*, Metabolite dosage, immunofluorescence, immunohistochemistry, in vivo studies, COMT expression analysis and statistical methods are described in the supplemental Materials and Methods.

## Results

### EAPB02303 inhibits PDAC cell viability and tumor growth

**Fig. 1:**
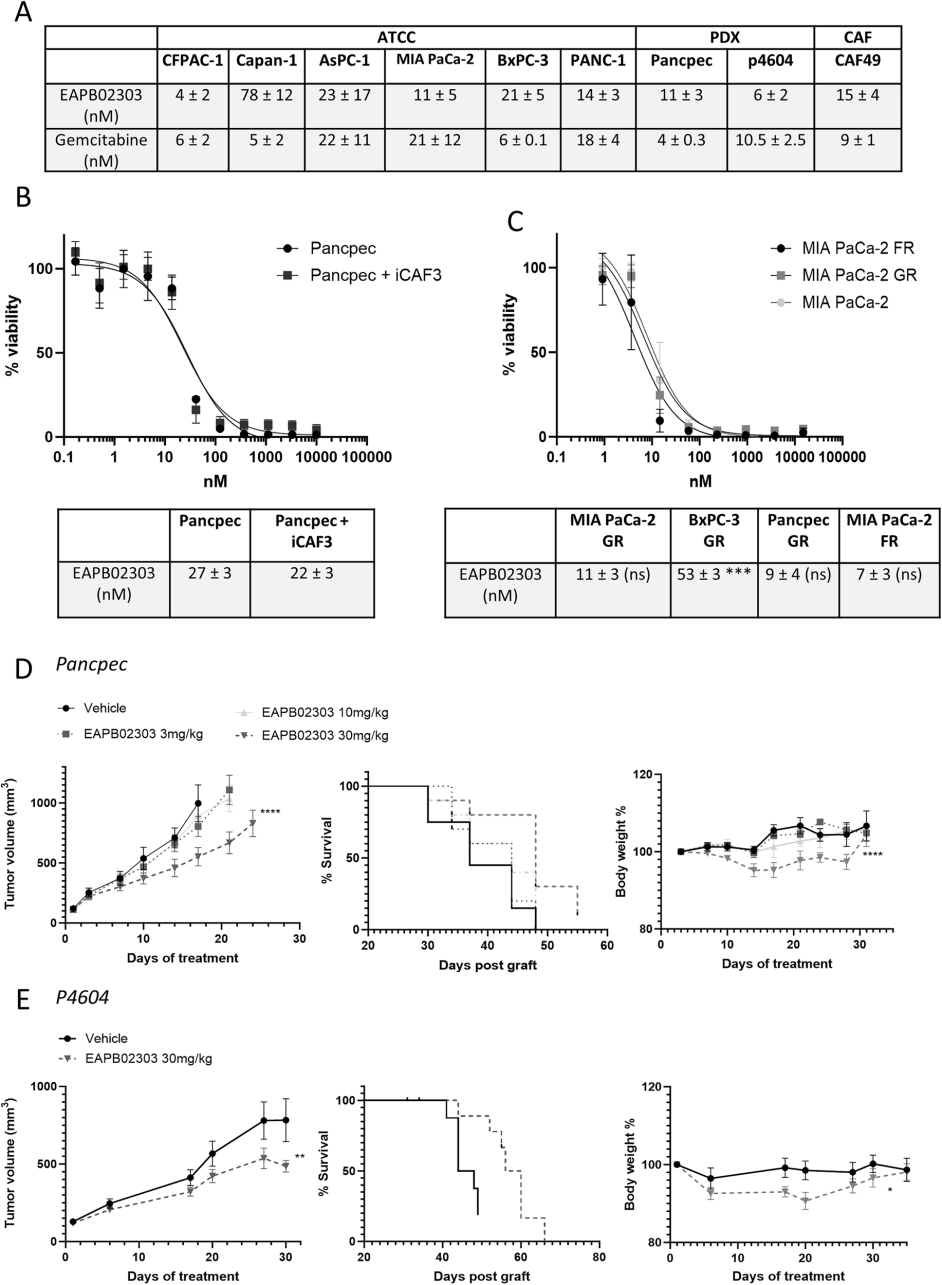
EAPB02303 alone affects pancreatic cancer cell viability in vitro and reduces tumor growth in vivo. EAPB02303 effect on cell viability was assessed using the SRB assay in the indicated PDAC cell lines (**A**), in 3D co-culture models with immortalized primary CAFs (ratio 1:50) (**B**), and in the indicated FOLFIRINOX-resistant (FR) and gemcitabine-resistant (GR) cell lines (**C**) (*n* = 3). Resistant cell line IC_50_ were compared to parental cell lines. Two tailed unpaired t-test p-values are reported ****p < 0.0001 ***p < 0.001, **p < 0.01, *p < 0.05. ns: not significant. Nude mice were xenografted with Pancpec (**D**) or P4604 (**E**) cells (*n* = 10 mice/group) and treated with EAPB02303 (3 mg/kg, 10 mg/kg, or 30 mg/kg) or vehicle (80% water, 0.9% NaCl, 10% DMSO, 10% Tween 20) 5 days/week for 30 days. Mouse weight and tumor size were measured throughout the experiment and Kaplan Meyer curves were computed. The significance is relative to the vehicle group. Error bars represents mean ± SEM. For tumor size and body weight, statistical testing using Linear Mixed-effect Models (LMMs) was performed, and p-values comparing the EAPB2303 30 mg/kg and vehicle groups are reported: ****p < 0.0001, ***p < 0.001, **p < 0.01, *p < 0.05, ns: not significant.

We first assessed the effect of EAPB02303 on the viability of various PDAC cell lines cultured alone (2D models) or with CAFs in 3D spheroid models (Fig. 1A, B). EAPB02303 IC_50_ ranged between 4 nM in CFPAC-1 and 78 nM in Capan-1 cells and was in the same range as that of gemcitabine in most of the tested cell lines (Fig. 1A). Co-culture of Pancpec tumor cells and CAFs (1:50 ratio) did not affect EAPB02303 cytotoxic effect (Fig. 1B). This is consistent with EAPB02303 ability to inhibit CAF viability (Fig. 1A). Moreover, EAPB02303 displayed significant cytotoxic effect in gemcitabine-resistant (GR) MIA PaCa-2 and Pancpec cells and also in FOLFIRINOX-resistant (FR) MIA PaCa-2 cells, as indicated by the IC_50_ values in the same range as those of the parental cell lines (*p* = 0.95, 0.49 and 0.46 respectively), except for BxPC3 cells (*p* = 0.003) (Fig. 1A, C).

We then assessed EAPB02303 in athymic mice xenografted with Pancpec or P4604 cells (subcutaneous injection). When tumor volume reached at least 150 mm^3^, we treated mice with EAPB02303 (3, 10, or 30 mg/kg) or vehicle alone every day for 30 days. EAPB02303 at 30 mg/kg significantly (*p* < 0.0001 in Pancpec and *p* = 0.0032 in P4604), but modestly reduced tumor growth. This led to an increase in overall survival (Pancpec: *p* = 0.012, P4604 *p* = 0.0064) (Fig. 1D, E). We observed significant weight loss following treatment with EAPB02303 at a dose of 30 mg/kg (Pancpec: *p* < 0.0001; P4604: *p* = 0.0153), which did not exceed 10% and tended to return to normal by the end of the treatment period. No other toxicities were observed.

These results demonstrated that EAPB02303 exerts a potent cytotoxic activity in vitro in PDAC cell lines with different mutational status (Supplementary Table 1) and reduces tumor growth in mice.

### Efficacy of the EAPB02303 and paclitaxel combination in preclinical PDAC models

**Fig. 2:**
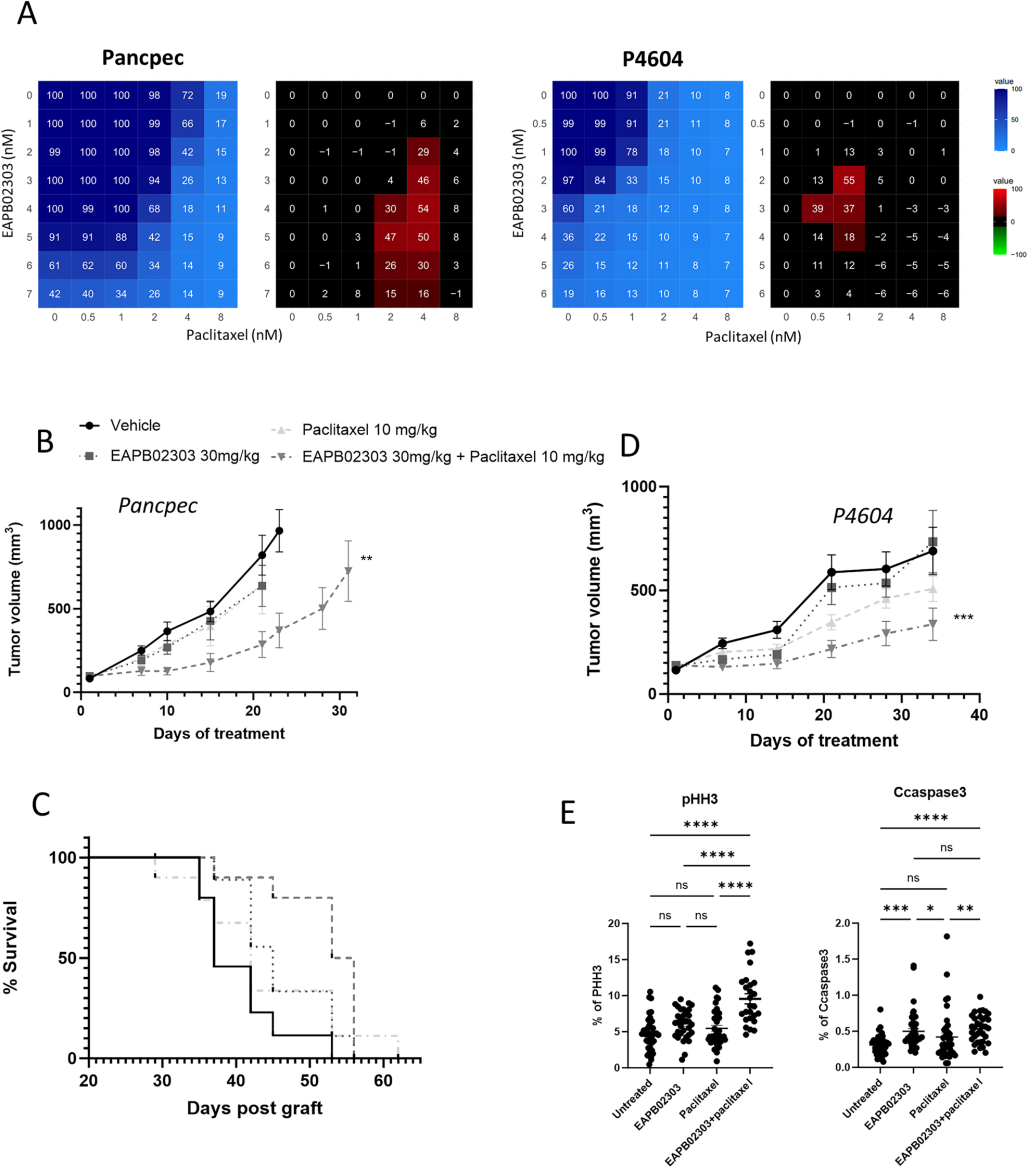
EAPB02303 displays synergy with paclitaxel in vitro and in vivo. **A** Cell survival (SRB assay) was assessed in Pancpec and P4604 cells after co-incubation with paclitaxel at increasing concentrations. The left matrices show the cell viability, expressed as percentage of survival, and the right matrices show the synergy, calculated as described in Materials and Methods. Blue: antagonism, black: additivity, red: synergy. Nude mice were xenografted (subcutaneous injection) with Pancpec (**B**, **C**) or P4604 (**D**) cells and treated with EAP02303 (30 mg/kg 5 days/week) or/and paclitaxel (10 mg/kg 2 days/week), combination, or vehicle (80% water, 0.9% NaCl, 10% DMSO, 10%Tween 20) (*n* = 10 mice/group). Tumor size was measured throughout the experiment and Kaplan Meyer curves were computed. For tumor size, statistical testing using Linear Mixed-effect Models (LMMs) was performed, and p-values comparing the combination treatment and vehicle groups are reported: ****p < 0.0001, ***p < 0.001, **p < 0.01, *p < 0.05, ns: not significant. **E** Expression of phosphorylated histone 3 (PHH3) and caspase 3 in P4604 cell tumors was analyzed at day 15 (mid-treatment) (*n* = 4). The mitotic and apoptotic index were calculated and represented as percentage. One-way ANOVA test was performed for the statistical analysis. Error bars represents mean ± SEM. ****p < 0.0001 ***p < 0.001, **p < 0.01, *p < 0.05. ns: not significant.

Then, we assessed the effect of EAPB02303 combined with clinically approved drugs for PDAC (Fig. 2A, Supplementary Fig. 1). The association of EAPB02303 with radiotherapy (Supplementary Fig. 1) was additive (black) in PDAC cell lines, while we showed a slight antagonism with gemcitabine (Supplementary Fig. 1). Surprisingly, the EAPB02303 and paclitaxel combination had a synergistic effect (red) in Pancpec and P4604 PDX-derived cells (Fig. 2A). This effect was confirmed in vivo, in mice xenografted with Pancpec and P4604 cells. The combination of EAPB02303 (30 mg/kg) and paclitaxel significantly inhibited tumor growth compared to vehicle in both models (*p* = 0.001 in Pancpec and *p* = 0.0007 in P4604) (Fig. 2B, D) and compared to each drug alone in Pancpec (*p* = 0.0224 for EAPB02303 and *p* = 0.0479 for Paclitaxel). Kaplan-Meier curves showed that survival was increased in mice treated with the combination compared with the vehicle or EAPB02303 alone in the Pancpec model (*p* = 0.008 and *p* = 0,0224 respectively) (Fig. 2C). Significant but modest weight loss (*p* = 0.0276) was observed in the Pancpec model following combination treatment, while no significant weight loss occurred in the P4604 model (Supplementary Fig. 2). We then analyzed by immunohistochemistry the drug effects on the expression of PHH3 (a mitosis marker, phosphohistone H3) and cleaved caspase 3 (an apoptosis marker) in tumors at mid-treatment (Fig. 2E, Supplementary Fig. 2). The treatment with EAPB02303 or Paclitaxel alone had no significant effect on PHH3 expression (mitotic index) compared to untreated mice (*p* = 0.1389 and *p* = 0.7643, respectively). In contrast, the combination treatment significantly increased PHH3 expression compared to both untreated cells and cells treated with either drug alone (*p* < 0.0001). The percentage of cleaved caspase-3-positive cells (apoptotic index) was significantly higher in tumors treated with EAPB02303 alone or in combination with Paclitaxel compared to untreated tumors (*p* = 0.0002 and *p* < 0.0001, respectively). However, Paclitaxel alone did not affect cleaved caspase-3 expression compared to untreated tumors (*p* = 0.9615). Notably, the combination treatment significantly increased cleaved caspase-3 expression compared to Paclitaxel alone (*p* = 0.0019), whereas EAPB02303 alone showed no significant difference (*p* > 0.99).

All these results demonstrated the potent cytostatic and cytotoxic activity of EAPB02303 in combination with Paclitaxel, both in vitro and in nude mice, leading to an increased mitotic index and enhanced apoptosis.

### The proteomic and transcriptomic profiles of EAPB02303-treated PDAC cells reveal interactions with mitosis regulation and microtubule dynamics

**Fig. 3:**
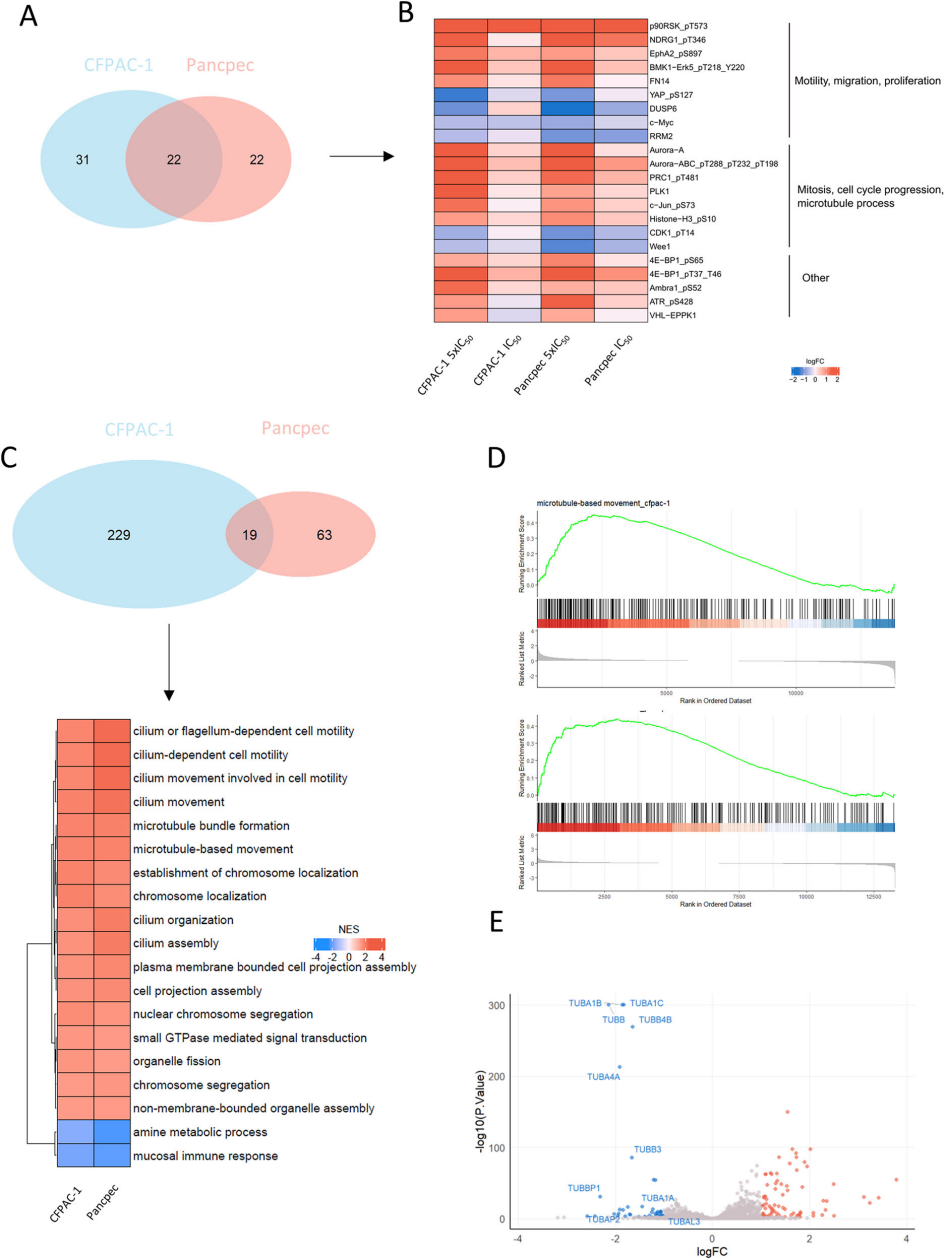
Proteomic and transcriptomic profile perturbations following incubation with EAPB02303. **A** Venn diagram showing the proteins differentially expressed or phosphorylated proteins (q < 0.05, logFC > 1.5) in Pancpec and CFPAC-1 cells after incubation with EAPB02303 (5xIC_50_) for 24 h compared with untreated controls (*n* = 3). **B** Heatmap showing the differentially expressed or phosphorylated proteins in the two cell lines after incubation with EAPB02303. **C** Venn diagram showing the Gene Ontology (GO) terms significantly enriched (q < 0.05) in the RNA-seq data of Pancpec and CFPAC-1 cells incubated with EAPB02303 (5xIC_50_) for 6 h compared with untreated controls (*n* = 3). The enrichment analysis was performed using GSEA and the GO Biological Process category from the Molecular Signatures Database (MSigDB). Heatmap (lower panel) of the GO terms significantly enriched in Pancpec and CFPAC-1 cells after incubation with EAPB02303. Many of the GO terms enriched in both cell lines were related to microtubule-based process. **D** Enrichment plots of the GO term “microtubule-based movement” in CFPAC-1 (upper panel) and Pancpec (lower panel) cells. **E** Volcano plot of the RNA-seq data of CFPAC-1 cells incubated or not with EAPB02303 (5xIC_50_ for 6 h) showing the high number of tubulin-encoding genes among the downregulated genes (q < 0.01, logFC > 1).

To elucidate EAPB02303 mechanism of action, we used transcriptomic and proteomic profiling. First, we evaluated the basal expression and phosphorylation levels of hundreds of proteins using the antibody-based RPPA in CFPAC-1 and Pancpec cells incubated or not with EAPB02303 (5xIC_50_ for 24 h). We identified 22 proteins that were differentially expressed or phosphorylated in both cell lines after incubation with EAPB02303 compared with untreated cells (Fig. 3A, B). Many of these proteins are implicated in cell cycle and cell division, especially in the control of mitosis: aurora A, B, C, PRC1, PLK1, JUN, histone H3, CDK1, WEE1 (Fig. 3B), suggesting a major effect of EAPB02303 on mitosis regulation.

We then carried out RNA-seq of Pancpec and CFPAC-1 cells incubated with EAPB02303 (5xIC_50_) or not for 6 h (Supplementary Fig. 4) or 24 h (data not shown) (three replicates). Incubation with EAPB02303 for 24 h massively altered the transcriptomic profiles of both cell lines, particularly apoptosis and stress-related genes. In cells incubated for 6 h, 51 and 10 genes were downregulated and 76 and 22 were upregulated in CFPAC-1 and Pancpec cells, respectively (Supplementary Fig. 4A) (LogFC > 1, q.value < 0.01). GSEA performed using the MSigDB subset GO: Biological process showed 229 and 63 significantly enriched gene sets in CFPAC-1 and Pancpec cells, respectively, among which 19 gene sets were shared by both cell lines (Fig. 3C). These 19 common gene sets are mainly related to processes involving microtubules (for instance, the gene set “microtubule based process”) (Fig. 3C, D). Incubation with EAPB02303 for 6 h led to the downregulation mainly of genes encoding tubulin isoforms or tubulin pseudogenes (Fig. 3E, Supplementary Fig. 4B). This suggests an autoregulatory mechanism in which excess free tubulin monomers negatively regulate tubulin transcription, a process that may be amplified following the disruption of microtubule polymerization, as previously reported [10]. Overall, these data suggest a major effect of EAPB02303 on microtubule dynamics and mitosis.

### EAPB02303 induces mitotic arrest and apoptosis

**Fig. 4:**
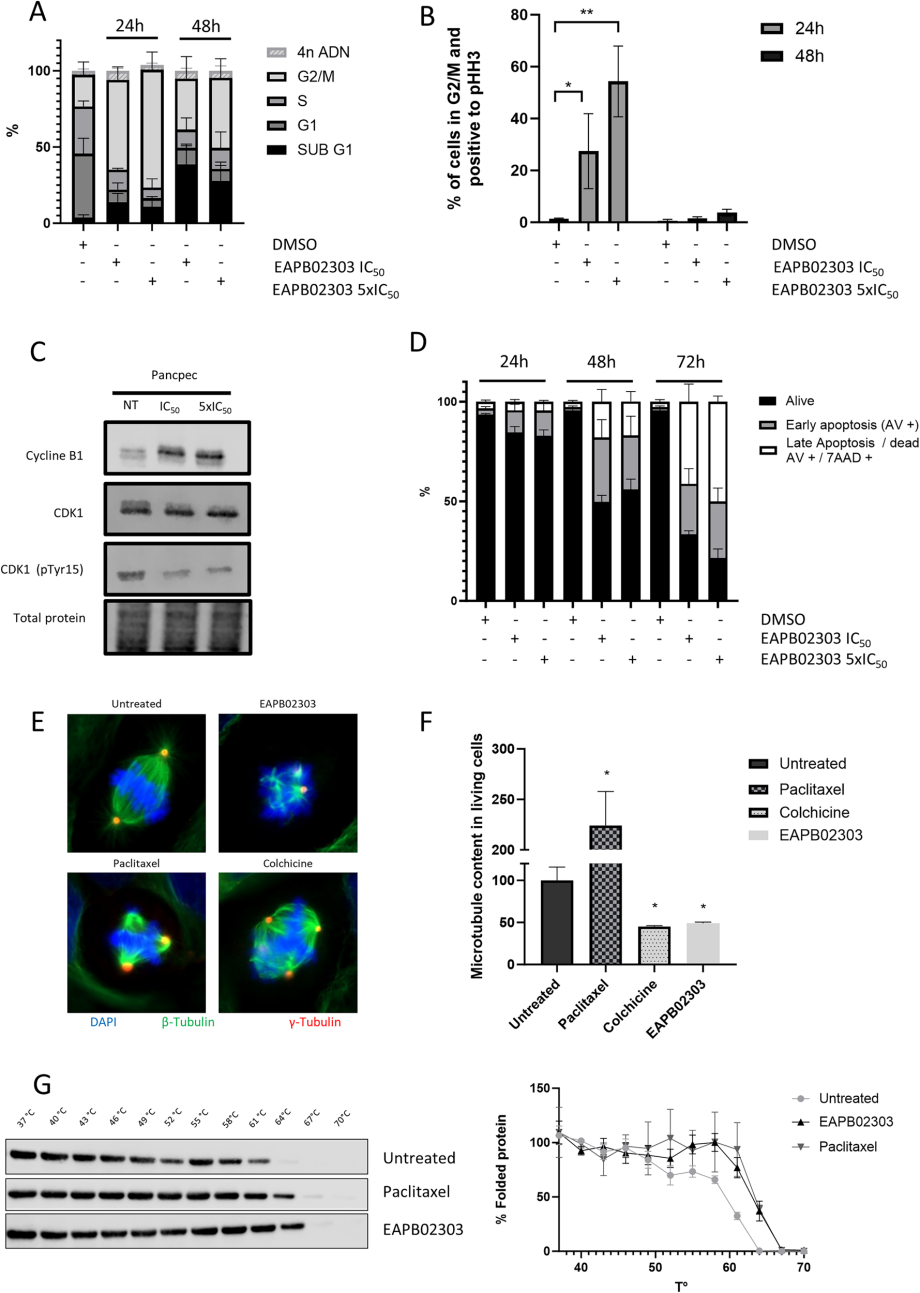
EAPB02303 affects cell cycle, induces apoptosis and alters microtubule polymerization in PDAC cells. Pancpec cells were incubated with EAPB02303 at the IC_50_ or 5xIC_50_ (IC_50_: 11 nM), fixed with 70% ethanol, stained with propidium iodide (**A**) and with an antibody against PHH3 (**B**) and analyzed by flow cytometry (*n* = 3). **C** Western blot analysis of key proteins of the G2/M checkpoint, cyclin B1, CDK1 and CDK1 phosphorylated at Tyr15, in Pancpec cells treated as described in A (*n* = 3). **D** Apoptosis was assessed by measuring Annexin 5/7-AAD staining by flow cytometry in Pancpec cells incubated with EAPB02303 (IC_50_ and 5xIC_50_) for 24, 48 and 72 h (*n* = 3). **E** CFPAC-1 cells were grown on coverslips and incubated with vehicle, EAPB02303, paclitaxel or colchicine, fixed with formaldehyde before staining with DAPI and antibodies against β-tubulin and γ-tubulin. Representative immunofluorescence images showing mitotic spindle disorganization following treatment with paclitaxel (6 h) and colchicine (24 h) (known MTAs) and also EAPB02303 (6 h). **F** Pancpec cells were incubated with vehicle, EAPB02303, paclitaxel or colchicine at 50 nM for 18 h. Cells were stained with an antibody against β-tubulin following permeabilization and fixation to allow the direct quantification of the microtubule biomass in cells. **G** CETSA melting curves of β-tubulin were generated using CFPAC-1 cell extracts after incubation with vehicle, EAPB02303 or paclitaxel for 3 h (*n* = 3). The shift in β-tubulin thermostability after incubation with EAPB02303 compared with vehicle indicates EAPB02303 engagement with β-tubulin. In panel change to Vehicle or Untreated. Error bars represents mean ± SD. Two tailed unpaired t-test p-values are reported ****p < 0.0001 ***p < 0.001, **p < 0.01, *p < 0.05. ns: not significant.

To experimentally confirm these findings, we performed a cell cycle analysis by propidium iodide staining and found that in PDAC cell cultures incubated with EAPB02303 (IC_50_ and 5xIC_50_), cells accumulated in the G2-M phase at 24 h and 48 h compared with untreated controls (Fig. 4A for Pancpec cells, Supplementary Fig. 5A for CFPAC-1 cells). Incubation with EAPB02303 for 48 h increased also the percentage of cells in the sub-G1 phase, suggesting that they cannot engage in the next cell cycle steps and that they will die.

Analysis of PHH3 (mitosis marker) showed an accumulation of Pancpec cells in mitosis only at 24 h (*p* = 0.035 for IC_50_ and 0.002 for 5xIC_50_) (Fig. 4B) and of CFPAC-1 cells in mitosis at 24 h and 48 h, associated with a typical rounded cells phenotype (Supplementary Fig. 5B, C). As the formation of a complex between CDK1 and cyclin B1 is an important event for mitosis entry, we assessed their expression in cells incubated or not with EAPB02303 (IC_50_ and 5xIC_50_) by western blotting. At 24 h, cyclin B1 expression was increased, whereas the expression of CDK1 phosphorylated at the inhibitory site Tyr15 was decreased (Fig. 4C), suggesting activation of the cyclin B1-CDK1 complex, in line with the accumulation of mitotic cells.

As drug-induced mitotic delay can lead to cell death [11], we investigated EAPB02303 effect on apoptosis induction by Annexin V and 7-AAD staining. In both Pancpec and CFPAC-1 cells, EAPB02303 (IC_50_ and 5xIC_50_) triggered early and late apoptosis already after 24 h of incubation. Apoptosis (early and late) rate reached 50% after 48 h in Pancpec cells and after 72 h in CFPAC-1 cells (Fig. 4D, Supplementary Fig. 5D).

These findings showed that EAPB02303 cytotoxic effect is achieved by blocking cells in mitosis and inducing apoptosis.

### EAPB02303 inhibits microtubule polymerization

As mitosis blockage is a common feature of agents that targets microtubule dynamics [12], we analyzed the effects on microtubule networks and cell morphology by immunofluorescence analysis of β-tubulin expression after incubation of cells with EAPB02303, colchicine, or paclitaxel for 6, 12 or 24 h. There are two major classes of microtubule-targeting agents (MTA) [13] : i) microtubule-stabilizing agents (e.g. taxanes and epothilones) bind to microtubule polymers and stabilize them against disassembly; ii) microtubule-destabilizing agents (e.g. colchicine and vinca alkaloids) bind to tubulin dimers and destabilize microtubule polymers [14]. As expected, colchicine and paclitaxel induced abnormal mitotic spindles with typical morphological changes, evoking destabilization (colchicine) or stabilization (paclitaxel) (Fig. 4E, Supplementary Fig. 5E). EAPB02303 also disrupted mitotic spindle formation in a dose-dependent manner (Fig. 4E, Supplementary Fig. 5E), yielding morphological changes similar to those observed with colchicine. This suggested that EAPB02303 could function as an inhibitor of microtubule polymerization. To test this hypothesis, we quantified the whole cell microtubule biomass after incubation with EAPB02303, colchicine, or paclitaxel for 18 h (Fig. 4F). Like colchicine, EAPB02303 led to a significant decrease in the quantity of cellular microtubules compared to untreated conditions (*p* = 0.0289), thus confirming its activity as microtubule polymerization inhibitor. Lastly, we used CETSA (Cellular Thermal Shift Assay) to determine whether this effect could be attributed to direct binding to tubulin. This assay relies on the principle of the thermal stabilization of the target (here, β-tubulin) upon drug binding directly in living cells. As expected, incubation with paclitaxel (a known tubulin binder) for 3 h increased the thermal stability of tubulin (*p* = 0.00007), as indicated by the increased tubulin detection at higher temperatures compared with untreated CFPAC-1 cells (Fig. 4G). We obtained similar results after incubation with EAPB02303 for 3 h (*p* = 0.001).

These results demonstrated that EAPB02303 inhibits microtubule polymerization, presumably through direct interaction with β-tubulin, as previously described for imidazo[1,2-*a*]quinoxaline compounds.

### Metabolization of EAPB02303 by catechol-O-methyl transferase (COMT)

**Fig. 5:**
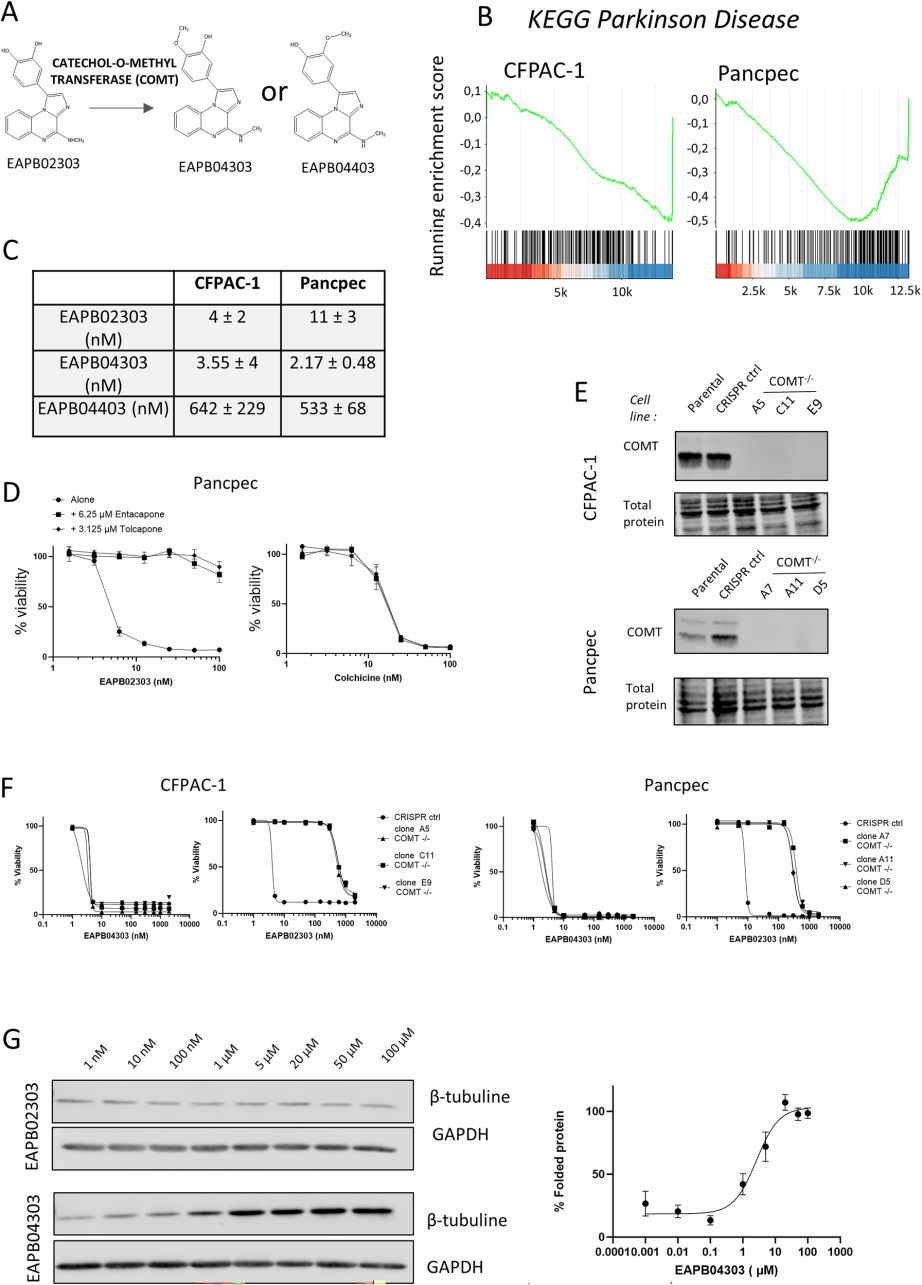
EAPB02303 is bioactivated by catechol-O-methyltransferase (COMT) to elicit its activity at nanomolar concentrations. **A** The hypothetical bioactivation of EAPB02303 by COMT would result in methylation at the 3’ or 4’ position of the catechol moiety, yielding EAPB04303 or EAPB04403. **B** Enrichment plots of the KEGG gene set “Parkinson disease” in Pancpec and CFPAC-1 cells after incubation with EAPB02303 (5xC_50_ for 6 h). **C** EAPB04303 and EAPB04403 were synthetized and their cytotoxicity in Pancpec and CFPAC-1 cells was assessed with the SRB assay and compared with that of EAPB02303 (*n* = 3). EAPB04303 was the most potent molecule with the lowest IC_50_, in line with the hypothesis that it is the active metabolite of EAPB02303. **D** CFPAC cells were incubated (or not) with the COMT inhibitors entacapone and tolcapone for 30 min before exposure to EAPB02303 or colchicine. The cytotoxic effect of EAPB02303, but not of colchicine, was assessed by SRB assay and abolished by the COMT inhibitors (*n* = 3). **E** The *COMT* gene was knocked out using the CRIPSR/cas9 system in CFPAC-1 and Pancpec cells and three different clones for each cell line were selected and validated by western blotting. **F** EAPB02303 activity was dramatically decreased in the three COMT^−/−^ clones of each cell line, while EAPB04303 activity remained unchanged. **G** ITDR-CETSA of β-tubulin showed that in CFPAC-1 COMT^−/−^ cells (clone E9), EAPB02303 cannot engage with tubulin, while EAPB04303 retains this activity.

As EAPB02303 does not inhibit microtubule polymerization when added to purified tubulin [4], we hypothesized that this discrepancy between purified tubulin and in-cell experiments might be explained by EAPB02303 bioactivation in cells. EAPB02303 has a catechol moiety that is an attractive candidate for bioactivation by COMT (Fig. 5A) to produce a hydro-methoxyphenyl metabolite. The yielded molecules would include 3-hydroxy-4-methoxyphenyl, a functional group also involved in combretastatin A4 interaction with the colchicine binding site on tubulin [15]. Two mono-methoxy compounds could be considered following EAPB02303 metabolization by COMT: EAPB04303 (3-hydroxy-4-methoxyphenyl derivative) and EAPB04403 (4-hydroxy-3-methoxyphenyl derivative).

Moreover, the GSEA analysis within the KEGG pathway collection of the RNA-seq data of Pancpec and CFPAC cells incubated with EAPB02303 for 6 h identified six common gene sets among which Parkinson’s disease was negatively enriched (Supplementary Fig. 6 and Fig. 5B). Due to the major implication of catecholamine metabolism and COMT inhibitors in Parkinson’s disease management, these results prompted us to investigate EAPB02303 bioactivation by COMT. First, we synthetized EAPB04303 and EAPB04403 from EAPB02303 in one step using a Suzuki-Miyaura cross-coupling reaction with the corresponding boronic acid (Supplementary Fig. 7). Then, we measured their cytotoxic activities (SRB assay) in CFPAC-1 and Pancpec cells. One of the predicted metabolites, EAPB04303, had a lower IC_50_ than EAPB02303, while EAPB04403 was less potent (Fig. 5C).

Then, we determined whether blocking COMT activity using the COMT inhibitors tolcapone and entacapone affected EAPB02303 cytotoxic effect (Fig. 5D). EAPB02303 cytotoxic activity was strongly reduced by COMT inhibition with these two inhibitors. Conversely, COMT inhibition did not affect colchicine cytotoxicity, underscoring the specificity of COMT dependency in EAPB02303 mechanism of action. To confirm these results and avoid any conclusions based on off-target effects of the pharmacological inhibitors, we generated Pancpec and CFPAC-1 cell lines in which COMT was knocked out using the CRISPR/cas9 technique (Fig. 5E). In the negative control (Pancpec and CFPAC-1 cells transduced with CRISPR luc), COMT expression was similar to that of parental cells (Fig. 5E). In line with our previous results, EAPB02303 cytotoxic effect was drastically reduced in cells lacking COMT (COMT^−/−^), as indicated by the up to 100 times higher IC_50_ (Fig. 5F, Supplementary Fig. 8A). Conversely, the predicted metabolite EAPB04303 displayed its potent cytotoxic effect in both COMT^−/−^ and COMT^+/+^ cell lines (Fig. 5F). To determine whether bioactivation by COMT was required for EAPB02303 binding to tubulin in cells, we performed ITDR-CETSA in CFPAC-1 COMT^−/−^ cells incubated with increasing concentrations of EAPB02303 or EAPB04303. By heating cells at 64 °C, a temperature that induces significant tubulin denaturation, ITDR-CETSA allowed calculating the EC_50_ value at which each molecule stabilized tubulin. Unlike in COMT^+/+^ cells, in COMT^−/−^ cells EAPB02303 could not induce tubulin stabilization even at the highest concentration tested (100 µM). Conversely, EAPB04303 had an EC_50_ of 3 µM ± 2 (Fig. 5G). This demonstrated that in cells, EAPB02303 engagement with tubulin is possible after its bioactivation by COMT, and suggests that in cells, EAPB02303 cellular activity on microtubule dynamics at nanomolar concentrations is due to binding of its active metabolite EAPB04303 to β-tubulin (Fig. 5G).

**Fig. 6:**
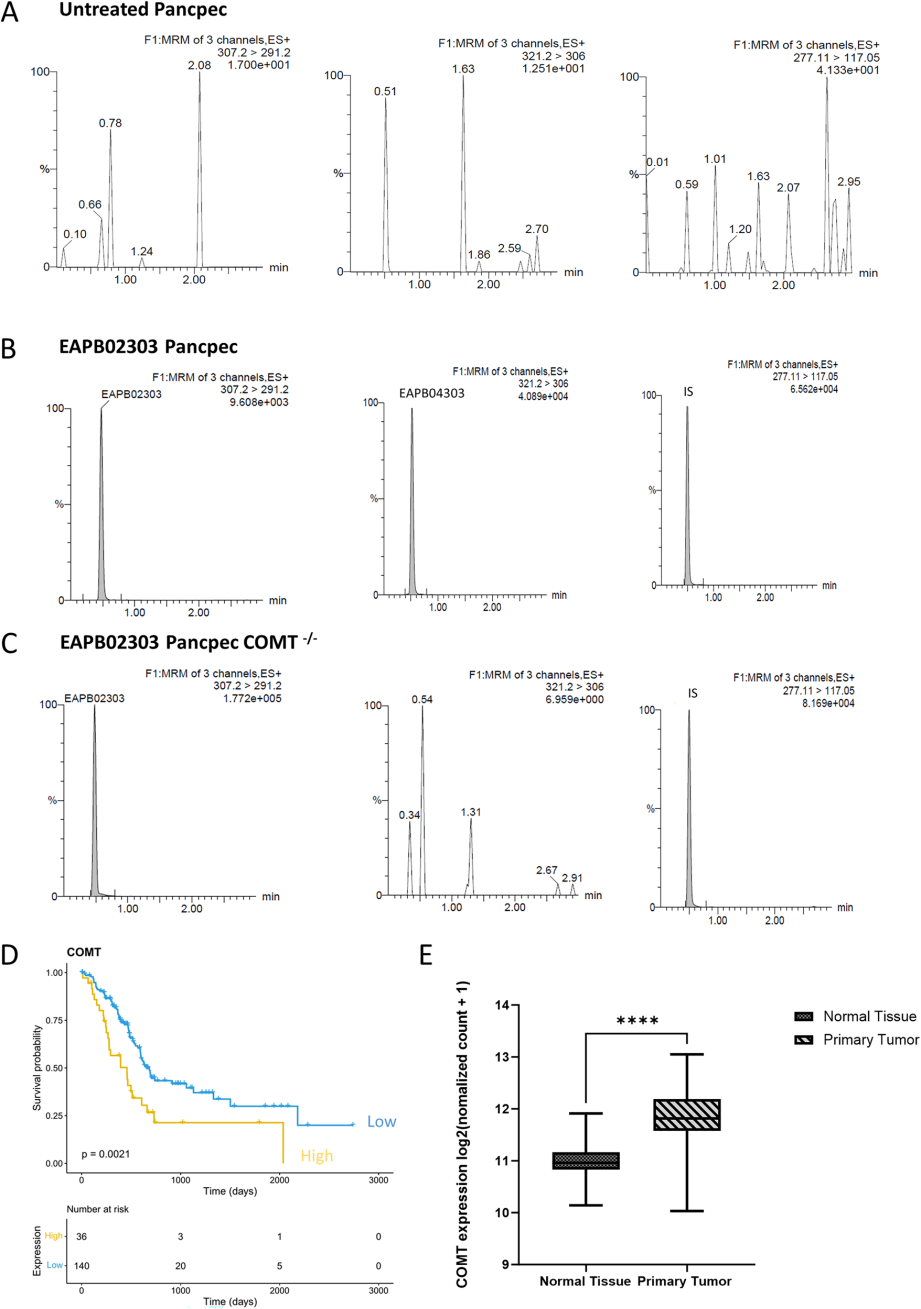
EAPB02303 is rapidly converted into EAP04303 in cells by COMT, an enzyme associated with poor prognosis in PDAC. Representative MRM chromatograms of EAPB02303 (307.2 → 291.2), EAPB04303 (321.2 → 306.0), and internal standard (IS) (277.1 → 117.1) in (**A**) untreated Pancpec cells, (**B**) Pancpec cells after incubation with 100 nM EAPB02303 after 60 min, and (**C**) Pancpec COMT^−/−^ cells after incubation with 100 nM EAPB02303 (**D**) Patients survival curves in function of *COMT* expression level (high/low) in pancreatic cancer samples. RNA-seq and survival data were from the TCGA database and were downloaded from the Human Protein Atlas. **E** *COMT* expression levels in normal pancreas and primary pancreatic cancer tissue samples from the TCGA and GTEx databases. Error bars represents mean ± SD. **** Two tailed unpaired t-test p-values are reported ****p < 0.0001 ***p < 0.001, **p < 0.01, *p < 0.05. ns: not significant.

We then used LC-MS/MS to measure the presence and concentration of EAPB02303 and its predicted active metabolite, EAPB04303, in both parental and COMT^−/−^ PancPec cells. The cells were incubated with 100 nM EAPB02303, either in the presence or absence of the COMT inhibitor entacapone (6.25 µM). After 15 and 60 min of incubation, we measured the drug concentration in cell extracts. Representative MRM chromatograms of EAPB02303 (307.2 → 291.2), EAPB04303 (321.2 → 306.0), and IS (Internal Standard) (277.1 → 117.1) are in Supplementary Fig. 8B, C. In untreated parental Pancpec cells, EAPB02303 and EAPB04303 were undetectable (below the limit of quantification, LoQ, of 0.49 ng/mL) (Fig. 6A). In parental Pancpec cells incubated with EAPB02303 for 60 min, the concentrations of EAPB02303 and EAPB04303 were 3.5 ± 0.7 ng/mL and 22.4 ± 1.7 ng/mL, respectively, confirming the production of the predicted metabolite (Fig. 6B).

In COMT^−/−^ Pancpec cells incubated with 100 nM EAPB02303 for 60 min, EAPB02303 concentration was 9.4 ± 0.8 ng/mL and EAPB04303 was undetectable (Fig. 6C). Similarly, after 60 min of incubation with EAPB02303 (100 nM) and entacapone (6.25 µM), the concentration of EAPB02303 in parental Pancpec cells was 6.6 ± 1.1 ng/mL, while EAPB04303 was undetectable (<LoQ) (Supplementary Fig. 8D).

Altogether, these data demonstrated that in PDAC cells, EAPB02303 is effectively and rapidly transformed into EAPB04303 by COMT, supporting the hypothesis that EAPB04303 is the actual microtubule-interacting agent responsible for EAPB02303 cytotoxic activity.

### COMT expression in PDAC samples

Analysis of data from the TCGA database on human PDAC samples (*n* = 176) showed a significant survival increase of patients with PDAC with low COMT mRNA expression (*p* = 0.0021) (Fig. 6D). Moreover, comparison of data from the TCGA and GTEx databases showed higher COMT expression in PDAC than normal pancreas tissue samples (*p* < 0.001) (Fig. 6E). These findings indicate that COMT could be used as biomarker to identify patients with PDAC who may respond to EAPB02303.

## Discussion

Despite many clinical studies, modest therapeutic gains have been observed in PDAC treatment as it remains one of the most lethal human solid tumors with a 5-year survival rate of 12% for all stages combined. Two chemotherapy combinations have been recently approved (gemcitabine with *nab-*paclitaxel, and FOLFIRINOX), but the response remains low [8, 16]. Therefore, the development and assessment of a novel and more specific therapeutic compounds that may be associated with the standard-of-care treatments is a challenge for translational research.

Here, first, we demonstrated that EAPB02303 at nanomolar concentrations can decrease cell viability in many PDAC cell lines (derived from tumors and PDX) to a similar extent as standard chemotherapy compound, such as gemcitabine. Our in vitro experiments showed that EAPB02303 has cytotoxic effects also in CAFs, one of the major components of PDAC microenvironment, and retained its effect in 3D models composed of tumor cells and CAFs. This is a crucial finding because of the high CAF proportion in the tumor mass and their involvement in chemoresistance.

We also elucidated the cellular and molecular mechanisms of EAPB02303 cytotoxicity. Using RNA-seq and RPPA, we found that EAPB02303 disrupts processes related to cell division, particularly mitosis. GSEA analysis revealed a strong enrichment of gene sets linked to microtubule-based processes and an over-representation of genes encoding tubulin isoforms among the downregulated genes. Recently, RNA-seq analysis of cell lines incubated with 1,3,4-oxadiazole chalcogen analogs gave similar results and contributed to the identification of these molecules as microtubule polymerization inhibitors [17]. Our RPPA results highlighted the differential expression and phosphorylation levels of proteins involved in mitosis execution in EAPB02303-treated PDAC cells compared with untreated controls. We experimentally confirmed these results by showing that upon exposure to EAPB02303, cells accumulated in mitosis followed by apoptosis. Moreover, by flow cytometry we showed that EAP02303 decreased the cellular microtubule content. This approach has been already successfully used to identify compounds that alter microtubule polymerization through direct interaction with tubulin. It is important to note that this approach could theoretically reveal microtubule polymerization inhibition by indirect mechanisms unrelated to direct interaction with tubulin, although this has never been described in the literature to our knowledge. Moreover, combination of similar RNA-seq analysis and whole cell microtubule analysis by flow cytometry was previously used to identify PTC596 as a new inhibitor of microtubule inhibition [18].

Microtubules targeting agents (MTAs) belong to a successful class of anticancer agents that have been used, particularly for breast, non-small cell lung and pancreatic cancer, in the last two decades [19, 20]. Most MTA binding sites are located in β-tubulin and only the pironetin site is in α-tubulin. In our study, CETSA assay showed β-tubulin shift in melting temperature upon EAPB02303 treatment. Previous work showed the efficiency of CETSA to assess target engagement of molecules targeting taxanes and vinca sites on β-tubulin [21]. Nevertheless, one limitation of such approach lies in the phenomenon of Thermal Proximity Coaggregation (TPCA) [22], which indicates that shifts in melting curves of a group of interacting proteins often correlates. Therefore, a careful analysis of our CETSA results can only confirm that EAPB02303 directly interacts with β-tubulin or one of its associated proteins. Taken together, these results highlight that EAPB02303 cytotoxic effect is mediated through mitotic arrest, mainly due to interaction with microtubule dynamic.

By binding and perturbing microtubule dynamics during mitosis, MTAs activate the spindle-assembly checkpoint and causes mitotic cell death [23]. However, their broad toxicity and pharmacological properties have limited their clinical use. Eribulin (approved for breast cancer) and *nab-*paclitaxel (approved for pancreatic cancer) are two advances in this drug class [24]. Eribulin inhibits microtubule polymerization and then extension without affecting microtubule shortening through non-competitive binding to the vinca alkaloid binding site and sequestration of tubulin into aggregates [25]. This unique mechanism is associated with reduced toxicity compared with vinca [26]. In *nab-*paclitaxel, paclitaxel is bound to nanoparticle albumin to improve its solubility, biodistribution and stability in circulation. Moreover, in the last decade, various tubulin-targeting antibody-drug conjugates have been marketed for patients with breast, urothelial or ovarian cancer and are currently used in clinical practice [27]. These examples demonstrate that novel MTAs could play an important role in oncology if they offer new/improved biochemistry and pharmacokinetic profiles.

Nevertheless, as patient response is highly variable and drug resistance remains a major clinical issue, combination therapies are increasingly used. Our findings indicated that EAPB02303 is efficient also in PDAC cell lines with acquired resistance to gemcitabine or FOLFIRINOX and therefore, could provide a second-line option in patients not responding to treatment. We also showed that the EAPB02303 and paclitaxel combination was synergic in several PDAC cell lines and also in two PDX-derived cell lines. Currently *nab-*paclitaxel is included in the standard treatments for PDAC, therefore, its combination with EAPB02303 could confer additional benefit. As these two MTAs may differently affect microtubule functions, this could delay the appearance of resistance. Combinations of two MTAs (e.g. paclitaxel and vinorelbine) have already been tested in preclinical and early clinical studies in different cancer types, for instance breast cancer [28]. Recently, a phenotypic screening highlighted synergistic effects between paclitaxel and carba1, which is an inhibitor of microtubule polymerization that targets the colchicine binding site of tubulin [29]. This highlights that MTAs with opposite mechanisms of action can achieve synergistic effects, and demonstrated the potential of such combinations to increase treatment efficiency and reduce toxicity.

Previous studies reported that EAPB02303 cannot inhibit microtubule polymerization when using purified tubulin. Here, we showed that EAPB02303 needs to be bioactivated by COMT to the mono-methoxy compound EAPB04303, which is responsible for EAPB02303 potent cytotoxic effect and its ability to disturb microtubule dynamics. By UPLC-MS/MS analysis of cellular extracts, we found that EAPB02303 is rapidly transformed into EAPB04303 by COMT. In agreement, when COMT was absent or inhibited, EAPB02303 was not cytotoxic at nanomolar concentrations and did not engage with β-tubulin. Conversely, EAPB04303 activity was not affected by COMT absence/inhibition. To our knowledge, this is the first report of a prodrug activated by COMT in the context of anti-cancer treatment.

COMT is a phase II enzyme that metabolizes endogenous (catecholamines, hormones) and exogenous (e.g. isoprenaline, dobutamine, levodopa) compounds carrying the catechol moiety, generating mainly inactive and hydrophilic compounds. It catalyzes O-methylation by transferring a methyl group to one of the hydroxyls of molecules carrying the catechol function [30]. This activity takes place mainly in the liver, but COMT is expressed at different levels in all body tissues, including kidney, brain and breast. COMT is mainly implicated in deactivation of neurotransmitters, such as dopamine and epinephrine. It is a well-known target in Parkinson’s disease, where the COMT inhibitors entacapone, tolcapone and opicapone are used in combination with levodopa to increase its half-life and potentiate its effect. Few studies have investigated the potential role of COMT in cancer management. Our survival analysis using TCGA data of patients with PDAC showed a correlation between high COMT expression and lower survival. Similarly, Wu and colleagues reported higher COMT expression in PDAC compared with healthy tissues [31]. Other studies showed *COMT* overexpression in colorectal cancer [32], and glioma [30]. In this last study, COMT inhibition led to sensitization of glioblastoma cells to radiotherapy by impairing mitochondria homeostasis, strongly supporting its targeting to treat glioma. COMT upregulation in PDAC and other cancer types compared with healthy tissues suggests that EAPB02303 could be preferentially bioactivated at tumor sites, potentially enhancing its efficacy and reducing its toxicity [30–32]. More studies are necessary to explore it.

Collectively, these data support the preclinical development of EAPB02303 as a promising anticancer agent in the treatment of PDAC, demonstrating synergistic effects when combined with the standard-of-care agent paclitaxel and functioning as a bioactivated compound through overexpressed COMT, a unique feature among other anticancer agents. Moreover, association with vectorizing strategies, such as specific antibodies, could enhance in the future therapeutic benefit by focusing even more site delivery of our cytotoxic lead.

## Supplementary information

Supplementary Material and Methods

supplementary Figure 1

Supplementary Figure 2

supplementary Figure 3

supplementary Figure 4

Supplementary Figure 5

supplementary Figure 6

supplementary Figure 7

supplementary Figure 8

supplementary Table 1

supplementary Table 2

Western blots

## Data Availability

Expression profiles analyzed in this study were obtained from the TCGA and Genotype-Tissue Expression (GTEx) databases. All other raw data generated or analyzed in this study are available upon request from the corresponding author.
