# Supplementary material for: Inhibiting microtubule polymerization with EAPB02303, a prodrug activated by catechol-O-methyl transferase, enhances paclitaxel effect in pancreatic cancer models
Source: Cell Death Dis. 2025 Jun 9;16(1):441. doi: 10.1038/s41419-025-07747-1 (PMC12149313; doi:10.1038/s41419-025-07747-1)
Supplement: Supplementary file 1 — Supplementary Material and Methods [file 41419_2025_7747_MOESM1_ESM.docx]

**Cell lines and reagents**

All human PDAC cell lines were from ATCC (Rockville, MD, USA) and were cultured following the ATCC recommendations (Supplementary Table 1). All cell lines were authenticated (Eurofins Genomics) and routinely tested for mycoplasma contamination using the MycoAlert™ Mycoplasma Detection Kit (LT07-318, Lonza). The Pancpec and P4604 cell lines were derived from PDX of a primary tumor and peritoneum metastases of human pancreatic tumor specimens, respectively (PDX Platform, Institut de Recherche en Cancérologie de Montpellier). Gemcitabine-resistant and FOLFIRINOX-resistant PDAC cell lines were generated as previously described (10) Primary cancer-associated fibroblasts CAFs and Immortalized CAFs were isolated from human PDAC samples using the outgrowth method, as described previously (11).

**Chemicals, drugs and reagents**

Gemcitabine and paclitaxel were from the Montpellier Cancer Institute pharmacy. Colchicine was purchased from Sigma (C9754). Entacapone and tolcapone were purchased from Sigma-Aldrich (SML0654, SML0150). The antibodies used in this work are listed in Supplementary Table 1.

EAPB02303 has been prepared as described in Patinote and colleagues (4). EAPB04303 et EAPB04403 have been synthetized starting from 1-bromo-*N*-methyl-imidazo[1,2-*a*]quinoxalin-4-amine according to the Suzuki Miyaura cross-coupling protocol described in Patinote and colleagues. Syntheses and full characterizations for EAPB04303 and EAPB04403 are available in supplementary data (Supplementary Figure 7).

Starting materials and solvents for Imiqualines preparation were obtained from Sigma-Aldrich (Saint-Louis, MO, USA). Ultrapure water (18.2 MΩ/cm) was prepared with a Milli-Q Plus 185 system (Millipore Corporation).

**2D cell growth assay**

Cell growth was evaluated using the SulfoRhodamine B (SRB) assay, as described by Combes and colleagues (12). Briefly, cells were seeded in 96-well plates and 24 hours later, drugs were added in serial dilutions. Cells were incubated for 96 hours and the percentage of viable cells was calculated relative to the untreated controls and plotted as a function of the drug concentrations using the Prism software to determine the IC_50_ (concentration that inhibits cell growth by 50%). Irradiations were performed 24 hours after drug treatment using an X-ray irradiator (Xenx, Xstrahl, Experimental Radiotherapy Core Facility, IRCM, Montpellier, France). Single doses of indicated Gy were delivered at a dose rate of 2.7 Gy/min (225 kV and 13.6 mA). The synergistic potential of the combinations was evaluated as described previously (13).

**3D cell growth assay**

Spheroids (3D cultures) were generated by seeding cell suspensions (100 μL/well) at optimized densities (50-2500 cells/well) in ultra-low attachment 96-well round-bottom plates (Fisher Scientific). For spheroids with CAFs, a ratio of 1 tumor cell to 50 iCAF3 was used. After 2 days, spheroids were incubated with drugs and cell viability was assessed at day 8 using the CellTiter-Glo Luminescent Cell Viability Assay (Promega), according to the manufacturer's instructions. Luminescence was measured using a 1450 MicroBeta TriLux Luminescence Microplate Reader (Perkin Elmer).

**Flow cytometry**

For cell cycle analyses, cells were plated and after 24 hours they were incubated with drugs. At the selected time points, cells were detached and washed in ice-cold PBS, fixed in 70% ethanol, and stained in 100 µL of anti-phosphorylated histone H3 (PHH3) antibody at room temperature for 20 minutes. Then, cells were diluted in 500 µL of PBS to have a concentration of 10 µg/mL Propidium Iodide (PI) and 100 µg/mL RNAse A. Cell-cycle distribution and PHH3 signal were determined with a Gallios Cytometer (Beckman Coulter) and quantified using the Kaluza software (Beckman Coulter).

For apoptosis quantification, cells were seeded in 6-well plates and incubated with the indicated drugs for at least 24 hours. Cells were stained with FITC-labeled Annexin V and/or 7-Amino-Actinomycin D (7-AAD; Annexin V/7-AAD kit Beckman Coulter) and apoptosis was evaluated using a Gallios Cytometer and the Kaluza software (Beckman Coulter).

**Whole-cell microtubule analysis**

Whole-cell microtubule polymerization assays were carried out by adapting the protocol described by Morisson and Hergenrother in 2012 (14). Cells were plated in 6-well plates for 24 hours and incubated with drugs for 18 hours. Then, cells were detached, pelleted and 1 mL of permeabilization buffer (80 mM PIPES pH 6.8, 1 mM MgCl_2_, 5 mM EDTA, 0.1% Triton X-100) at room temperature was added for 10 minutes. After centrifugation at 1500 rpm for 5 minutes, cells were incubated with microtubule stabilizing buffer (80 mM PIPES pH 6.8, 1 mM MgCl_2_, 5 mM EDTA, 0.5% Triton X-100 and 0.5% glutaraldehyde) for 10 minutes. Glutaraldehyde auto- fluorescence was quenched by adding 0.7 ml of 1 mg/mL NaBH_4_ in PBS. Cells were pelleted, and the supernatant was removed by gentle aspiration. After a PBS wash, cells were incubated in 25 µL antibody diluting solution (PBS pH 7.4, 0.2% Triton X-100, 2% bovine serum albumin [BSA]) at 4°C for 1 hour, and then 0.5 µL of an anti-tubulin–FITC antibody was added to achieve the concentration of 1:50. Cells were incubated at 4°C in the dark for 3 hours, and then diluted in 200 µL PBS before transfer into flow cytometry tubes and analysis with a Gallios Cytometer (Beckman Coulter). The geometrical mean of the FITC channel was used to assess tubulin level that was normalized to the untreated condition (set to 100).

**Western blot analysis**

Cells were lysed and blotted as previously described (11). Immunoreactions were revealed using fluorescent-labeled secondary antibodies and were visualized with the Odyssey XF Imaging System (LI-COR). Bands were quantified with the Image Studio Lite software and data were relative to the total protein amount measured using the Revert™ 700 Total Protein Stain for Western Blot Normalization. The effects of drugs on protein expression were expressed as fold change relative to the expression level in the untreated condition (set to 1).

**Cellular thermal shift assay (CETSA)**

CETSAs were carried out by adapting the protocols described by Jafari and colleagues (15) and Langebäck and colleagues (16). For the protein melting curve analysis, cells were harvested, washed, and then, 500,000 cells were transferred into PCR tubes and pelleted. 100 µL of a 20 µM drug solution in PBS was added and cells were incubated at 37°C for 3 hours. Then, cells were heated using a Biometra Tri Analytik Jena thermocycler to the selected temperatures for 3 minutes. After heating, cells were immediately snap frozen in liquid nitrogen and stored at -80°C. Cells were then lysed by three cycles of freeze-thawing using liquid nitrogen and a thermocycler set at 20°C, before centrifugation at 15,000 g for 30 minutes. 80 µL of supernatant was collected and diluted in 4x Laemmli buffer, vortexed, heated at 95°C for 5 minutes and loaded in 10% acrylamide gels for protein separation by electrophoresis.

For the IsoThermal Dose Response (ITDR-CETSA), cells were harvested, washed, and incubated in PCR tubes at the concentration of 10 million cells/mL in 50 µL PBS. 50 µL of drugs was then added (2X concentration in PBS) and after 3 hours of incubation at 37°C cells were heated, using a Biometra Tri Analytik Jena thermocycler, to 64°C for 3 minutes, snap frozen in liquid nitrogen and processed as described for the melting curves.

**RNA sequencing (RNA-seq) and data analysis**

Total RNA from CFPAC-1 and Pancpec cells incubated or not with EAPB02303 for 6 or 24 hours, in triplicate, was extracted using the Quick-RNA Miniprep Kit (ZymoResearch #R1055). RNA quality (RIN) was assessed using a 2100-Bioanalyzer (Agilent Technologies) by the NGS Core Facility Platform (IRMB, Montpellier). RNA samples with a RIN >7 were sequenced by the Brain Institute Genotyping and Sequencing Core Facility (iGenSeq, Paris). mRNA libraries were prepared and sequenced as already described (17). The raw data quality was evaluated with FastQC (https://www.bioinformatics.babraham.ac.uk/publications.html). Poor quality sequences and adapters were trimmed or removed with the software fastp (18), using default parameters, to retain only good quality paired reads. The Illumina DRAGEN bio-IT Platform (v3.8.4) was used for mapping to the reference human genome hg38 and for quantification with the Gencode v37 annotation gtf file. Library orientation, library composition and transcript coverage were checked with the Picard tools. The following analyses were done with the R software. Data were normalized with the DESeq2 (19) packages before differential analysis using the glm framework likelihood ratio test from the DESeq2 workflow. Adjusted p-values for multiple hypotheses were calculated with the Benjamini-Hochberg procedure to control the false discovery rate (FDR). The enrichment analysis was performed with the clusterProfiler R package (20) and Gene Set Enrichment Analysis (GSEA) of the GO Biological Process and KEGG gene set collection (21).

**Reverse-phase protein array (RPPA)**

RPPA analysis was carried out by MD Anderson Cancer Center. CFPAC-1 and Pancpec cells were incubated or not with EAPB02303 at IC_50_ or 5xIC_50_ for 6 hours, in triplicate. After serial dilutions, protein extracts were spotted onto nitrocellulose-coated slides plus replicate controls. Specific antibodies were used to amplify the signal *via* a tyramide signal amplification system and were visualized by DAB (3,3’-diaminobenzidine) colorimetric reaction to detect the proteins of interest. Each slide was probed with one antibody. The detection system used was a GenPoint™ staining kit from Agilent. Digital images of slides were obtained by scanning them on a Huron TissueScope scanner that produced 16-bit TIFF files. Spot intensities from the TIFF files were determined with the Array-Pro Analyzer software. Differentially expressed proteins were identified based on the normalized data between control and treated samples using the limma package in R; a FDR < 0.01 and logFC > 1 were used as cutoff to draw volcano plots.

**CRISPR-Cas9-mediated knock-out of *COMT***

CFPAC-1 and Pancpec cells in which *COMT* was knocked out were generated using the COMT sgRNA CRISPR/Cas9 All-in-One Lentivector set (Human) (166181110595). CPFAC-1 and Pancpec cells were infected with retroviral particles and after 96 hours, transduced cells were selected by adding 2 µg/mL puromycin for 6 days. Cells were amplified and cloned in 96-well plates. After 10 days, positive clones were re-seeded and screened by western blotting for COMT knock-out.

**Metabolite dosage**

*In vitro* Pancpec cell sample preparation

Pancpec and Pancpec^COMT-/-^ cells were used to study the biotransformation of EAPB02303 and particularly into EAPB04303 after addition or not of the COMT inhibitor entacapone at 6.25 µM. Cells were seeded for 24 hours and incubated with free or 6.25 µM entacapone culture medium for 30min prior to addition of EAPB02303 at 100 nM and incubation for 60 minutes. Cells were detached using a scraper in ultrapure water to obtain a suspension of partially lysed cells. Cell suspensions were sonicated to complete cell lysis, followed by centrifugation at 14,000 g for 15 minutes. Supernatants were frozen in liquid nitrogen before analysis by liquid chromatography-tandem mass spectrometry (UPLC-MS/MS). For each condition, five technically independent samples were analyzed.

*Quantification of EAPB02303 and EAPB04303 by LC-MS/MS analyses*

Stock solutions, working solutions, calibration curves and sample preparation were prepared as already described (5). All measurements were performed on an Acquity UPLC I-Class Plus System (Waters, Milford, USA) linked with a Waters Xevo TQD tandem triple quadrupole mass spectrometer. The chromatographic separation was conducted by injecting 5 µL of each calibration standard solution or *in vitro* sample on an Acquity UPLC BEH C18 column (1.7 µm, 2.1 mm x 50 mm) from Waters at 40°C. A mixture of acetonitrile (eluent A) and ultrapure water (eluent B) (50/50,v/v) with 0.1% formic acid was used as mobile phase at a flow rate of 0.5 mL/min without split. All solvents were filtered through a 0.20 µm millipore filter (Molsheim, France) before use and degassed 15 min in an ultrasonic bath. The autosampler was set at 4°C. The MS/MS was operated in positive electrospray ionization (ESI+), with operating conditions as follows: capillary voltage at 3.0 kV, desolvation temperature at 500 °C, source temperature at 150 °C, desolvation gas flow at 500 L/h, and cone gas flow at 150 L/h. Detection of EAPB02303 and EAPB04303 was performed using Multiple Reaction Monitoring (MRM). The optimal MRM transitions for precursor ion [M+H]+ to specific product ion were m/z 307.2 → 291.2 for EAPB02303 m/z 321.2 → 306.0 for EAPB04303, and m/z 277.1 → 117.1 for the internal standard (IS) (Fig 6.). Optimal MS/MS parameters (cone voltage and collision energy) for MRM transitions of EAPB02303, EAPB04303 and IS are 60V/50V, 60V/40V and 70V/60V respectively. The acquisition dwell time was 108 ms for all transitions. Mass spectra were collected in scan mode (m/z 50–1000). The LC–MS/MS system was controlled and the analytical data were collected and processed using MassLynx software version 4.2 (Waters).

**Immunofluorescence analyses**

Cells were plated on coverslips in 24-well plates. After 48 hours, cells were incubated with drugs for 6, 12 or 24 hours. After fixation with 3.7% formaldehyde in PBS for 20 minutes, cells were permeabilized with PBS/0.5% Triton X-100 at room temperature for 15 minutes. After washes, cells were incubated in PBS/2% BSA for 1 hour, and then with primary antibodies (1/500) (Supplementary Table 1) at 37°C for 90 minutes. Cells were washed twice with PBS/0.1% Tween-20 and were incubated with the goat anti-mouse IgG Alexa 488 (Cell Signaling #4408; 1/50) and goat anti-rabbit AF568 (Invitrogen A11011; 1/500) secondary antibodies at 37°C for 45 minutes. Then, coverslips were washed with PBS/0.1% Tween-20 three times and with PBS three times, followed by mounting with Everbrithe^®^ and DAPI and analysis using an epifluorescence Zeiss Axio Imager 2 microscope.

**Immunohistochemical analyses**

Immunohistochemistry analyses were performed as described previously (22). Signals were quantified with QuPath. Mitotic and apoptotic index were calculated as the ratio of the number of PHH3 or CCaspase 3 positive cells/ the number of total cells. The results are presented as the percentage of mitotic or apoptotic index.

***In vivo* studies**

All *in vivo* experiments were performed in compliance with the French regulations and ethical guidelines for experimental animal studies in an accredited establishment (Agreement No. C34-172-27). P4604 (5 x 10^6^) or Pancpec (5 x 10^6^) PDX-derived cells were grafted subcutaneously in 6-week-old female athymic mice, purchased from Charles Rivers (Le Malcourlet, France). Tumor-bearing mice were randomized in the different treatment groups (10 animals/group) when tumors reached a minimum volume of 150 mm^3^. Tumor volumes were calculated with the formula: D_1_ x D_2_ x D_3_ /2. Mice were treated by intra-peritoneal injection of EAPB02303 (30 mg/kg in vehicle: 80% isotonic water, 10% DMSO, 10% Tween 80) daily, and/or with paclitaxel (10 mg/kg) twice per week, or vehicle alone daily for 4 weeks. For survival comparison, mice were sacrificed when tumors reached a volume of 1500 mm^3^ or 1000 mm^3^ for the combination experiments. Animals were excluded of the experiment when a limit point has been reached (tumor size, loss of weight…). The treatment and tumor measurement were done blindly.

***COMT* expression analysis**

COMT expression in PDAC tissues and survival data were downloaded from the TCGA database and log-rank test was used to compare survival in COMT low and high groups of patient.

*COMT* expression data in tumor and normal pancreatic tissues were downloaded *via* the Xena platform from the TCGA and Genotype-Tissue Expression (GTEx) databases. Data were visualized as boxplots and expression levels in normal and tumor tissues were compared with the unpaired t-test using Prism v10.0.2.

**Statistical analysis**

Data were expressed as the mean ± SEM from at least three experiments, and the two-tailed Student’s t or 1-way ANOVA tests (for more than two groups) were used to calculates p-values. Statistical difference was set at *p ≤0.05, ∗∗p <0.01, ∗∗∗p <0.001, ∗∗∗∗p <0.0001. The relationship between tumor growth and treatment was analyzed using a linear mixed regression model. The fixed part of the model included the number of days post-graft and treatment group; interaction terms were also evaluated. Random intercepts were included to account for the variability in baseline tumor volumes between individual subjects. The model coefficients were estimated by maximum likelihood. A survival analysis was performed and the considered event was a tumor volume of 1000 mm^3^. Survival rates were estimated using the Kaplan Meier method and survival curves were compared with the log rank test. Statistical significance was set at p <0.05. Statistical analyses were done with R.
