# Supplementary figures and images for: Inhibiting microtubule polymerization with EAPB02303, a prodrug activated by catechol-O-methyl transferase, enhances paclitaxel effect in pancreatic cancer models
Source: Cell Death Dis. 2025 Jun 9;16(1):441. doi: 10.1038/s41419-025-07747-1 (PMC12149313; doi:10.1038/s41419-025-07747-1)

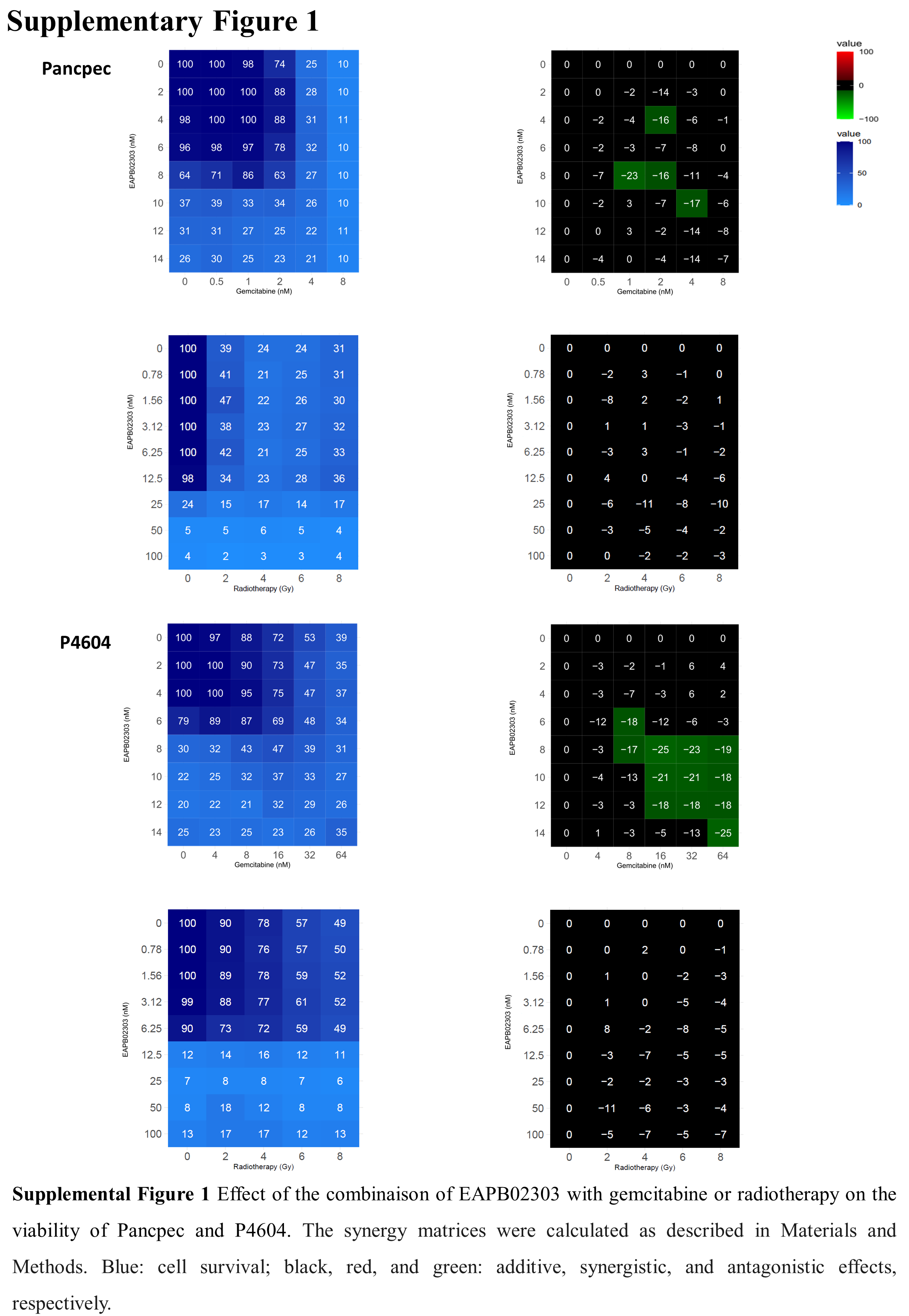

Supplement: Supplementary file 2 — supplementary Figure 1 [file 41419_2025_7747_MOESM2_ESM.tif]

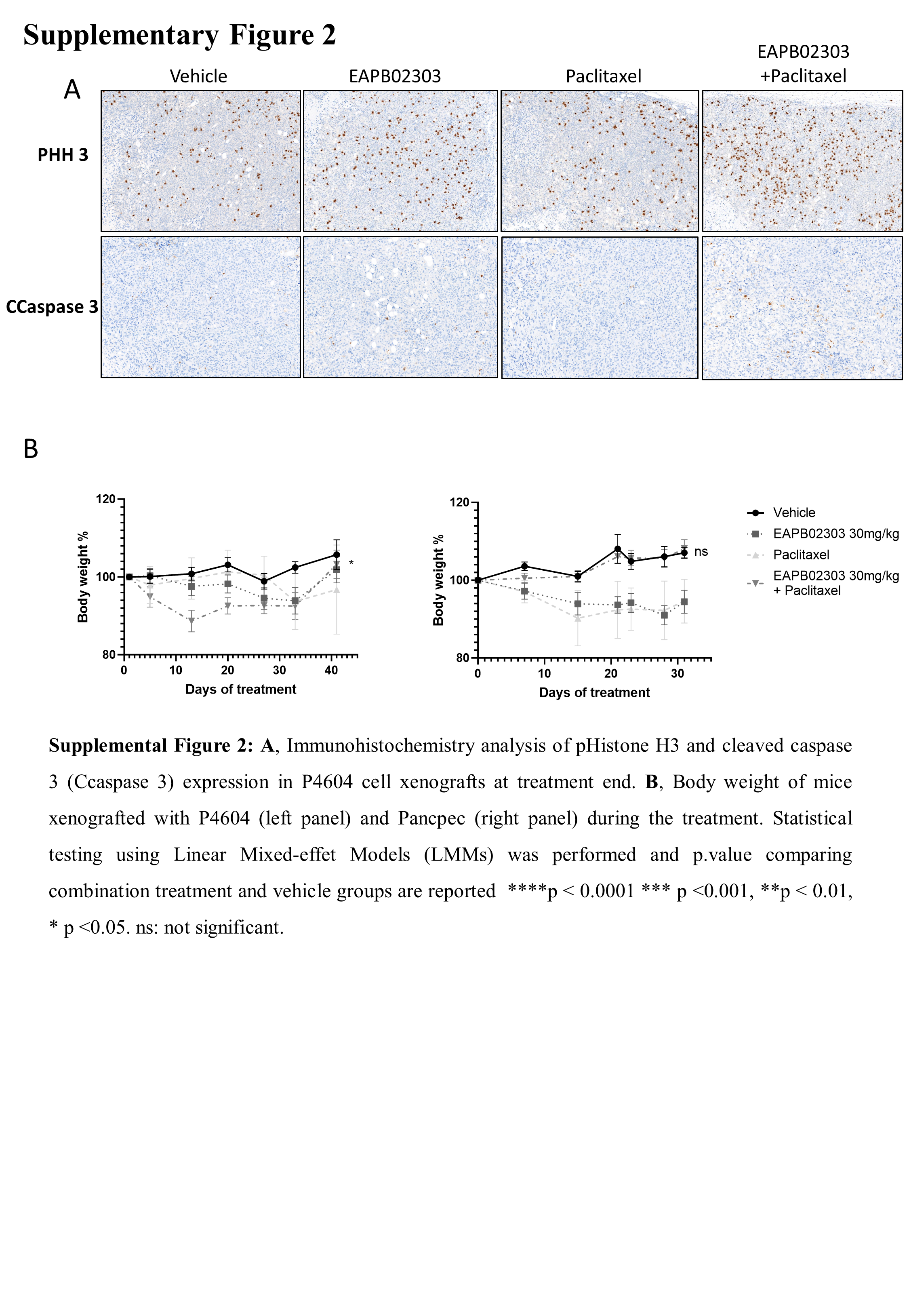

Supplement: Supplementary file 3 — Supplementary Figure 2 [file 41419_2025_7747_MOESM3_ESM.tif]

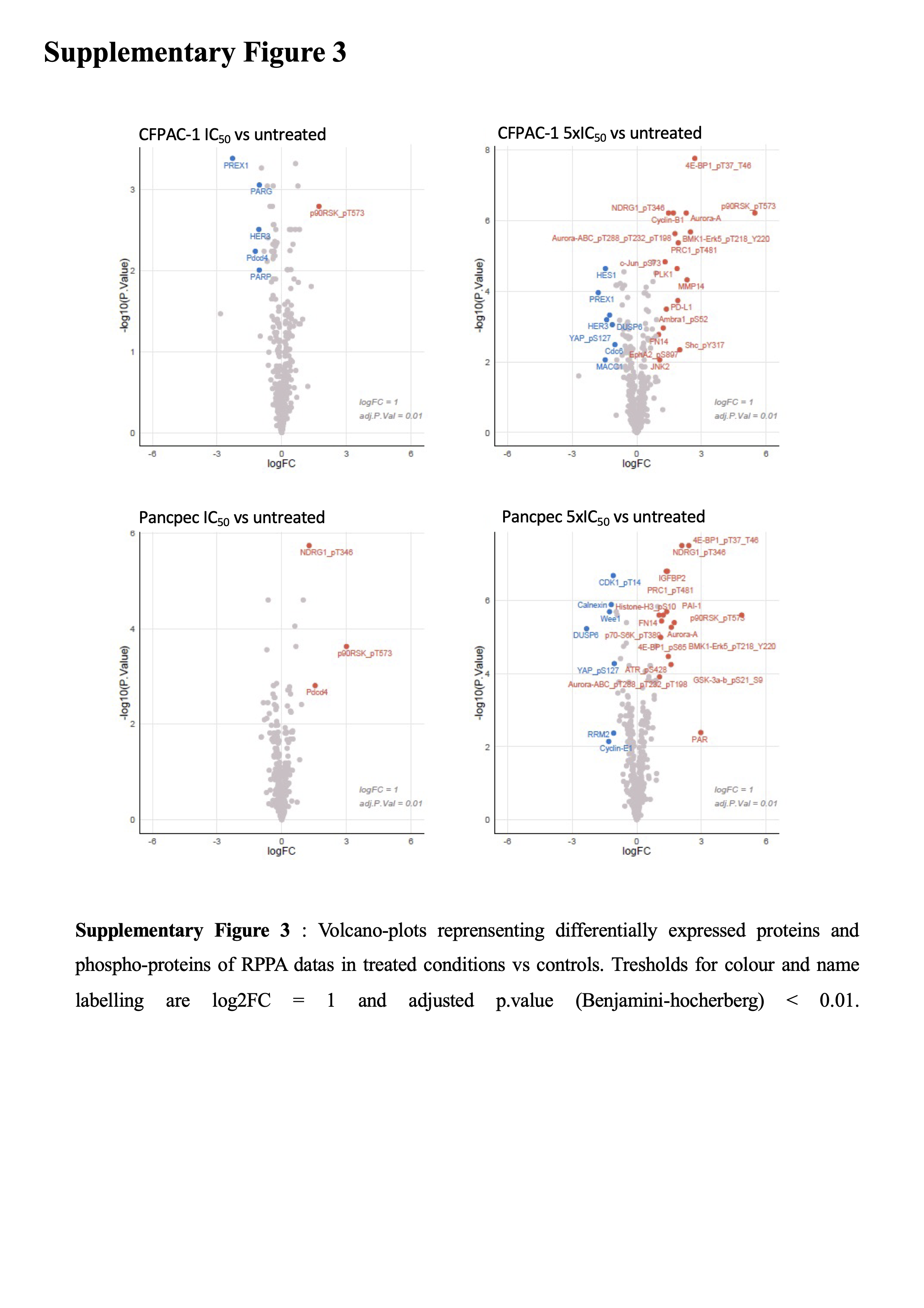

Supplement: Supplementary file 4 — supplementary Figure 3 [file 41419_2025_7747_MOESM4_ESM.tif]

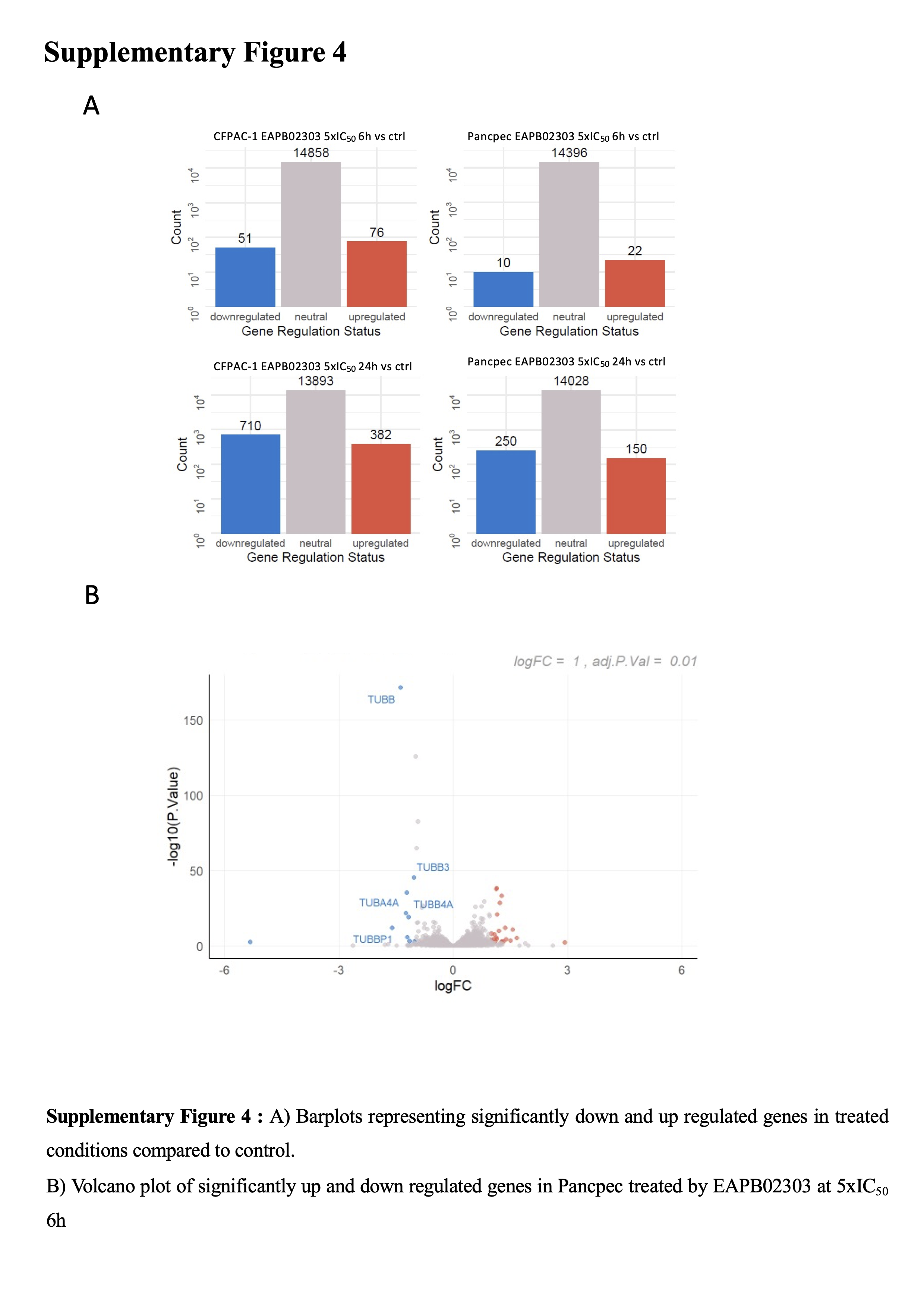

Supplement: Supplementary file 5 — supplementary Figure 4 [file 41419_2025_7747_MOESM5_ESM.tif]

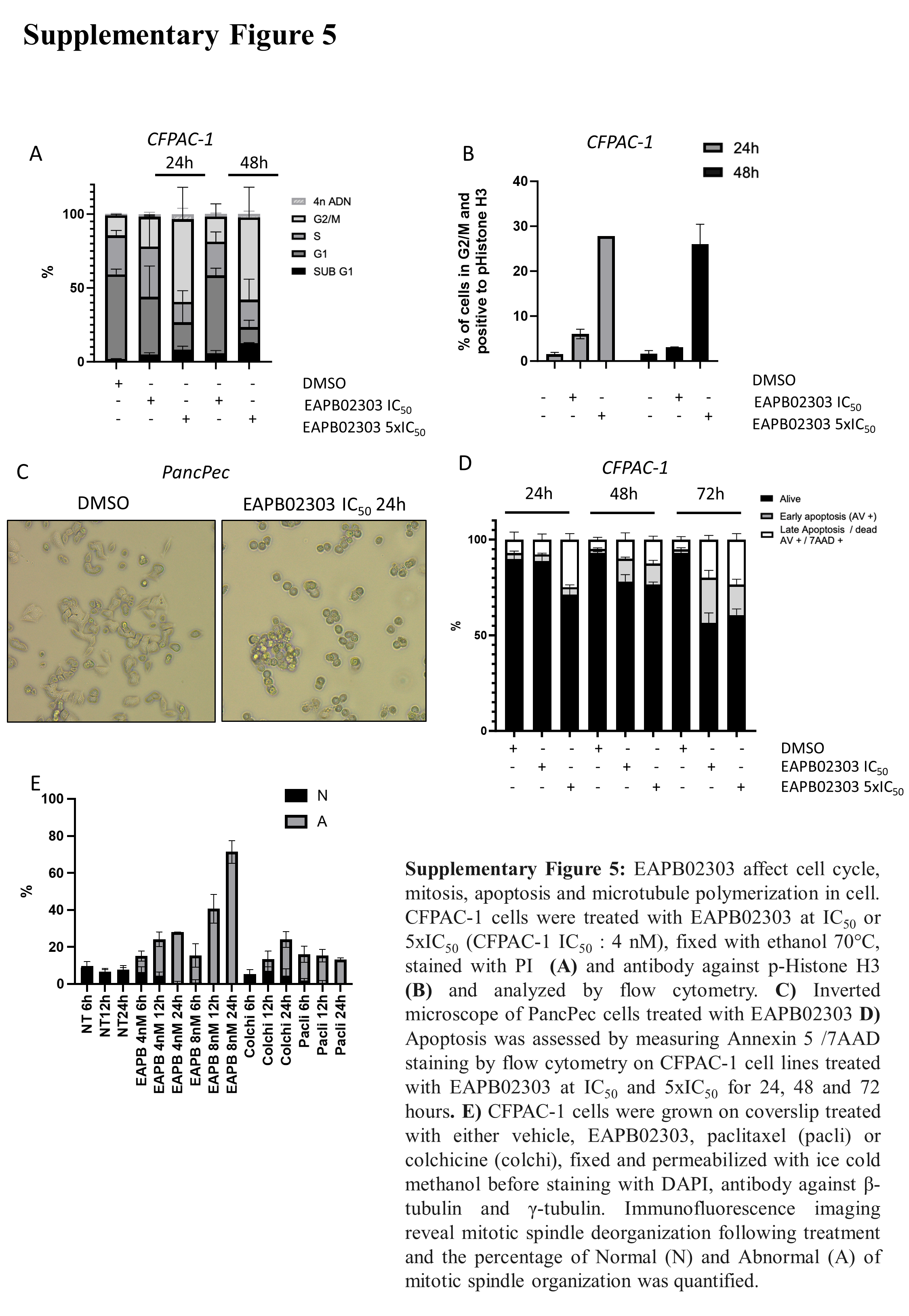

Supplement: Supplementary file 6 — Supplementary Figure 5 [file 41419_2025_7747_MOESM6_ESM.tif]

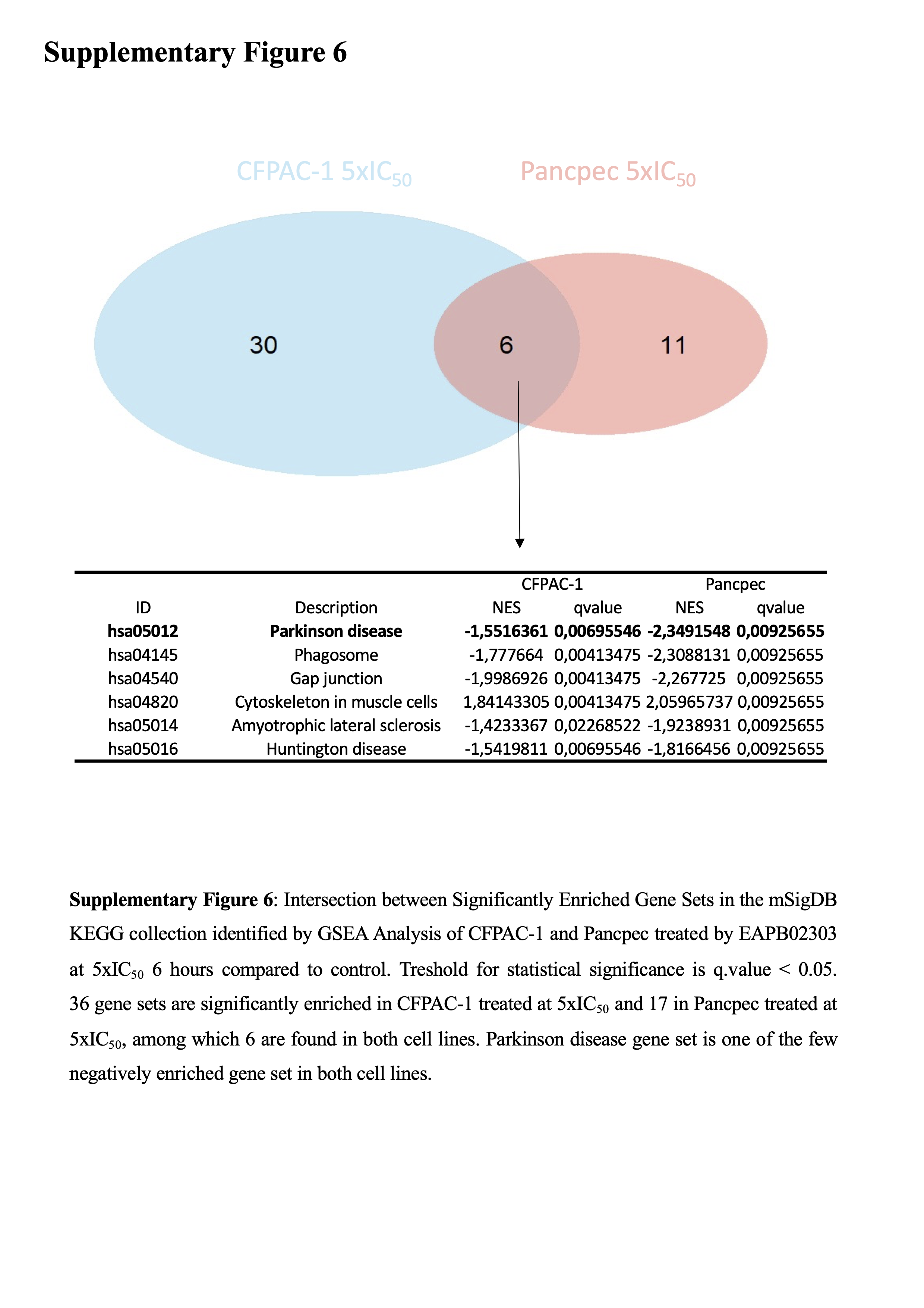

Supplement: Supplementary file 7 — supplementary Figure 6 [file 41419_2025_7747_MOESM7_ESM.tif]

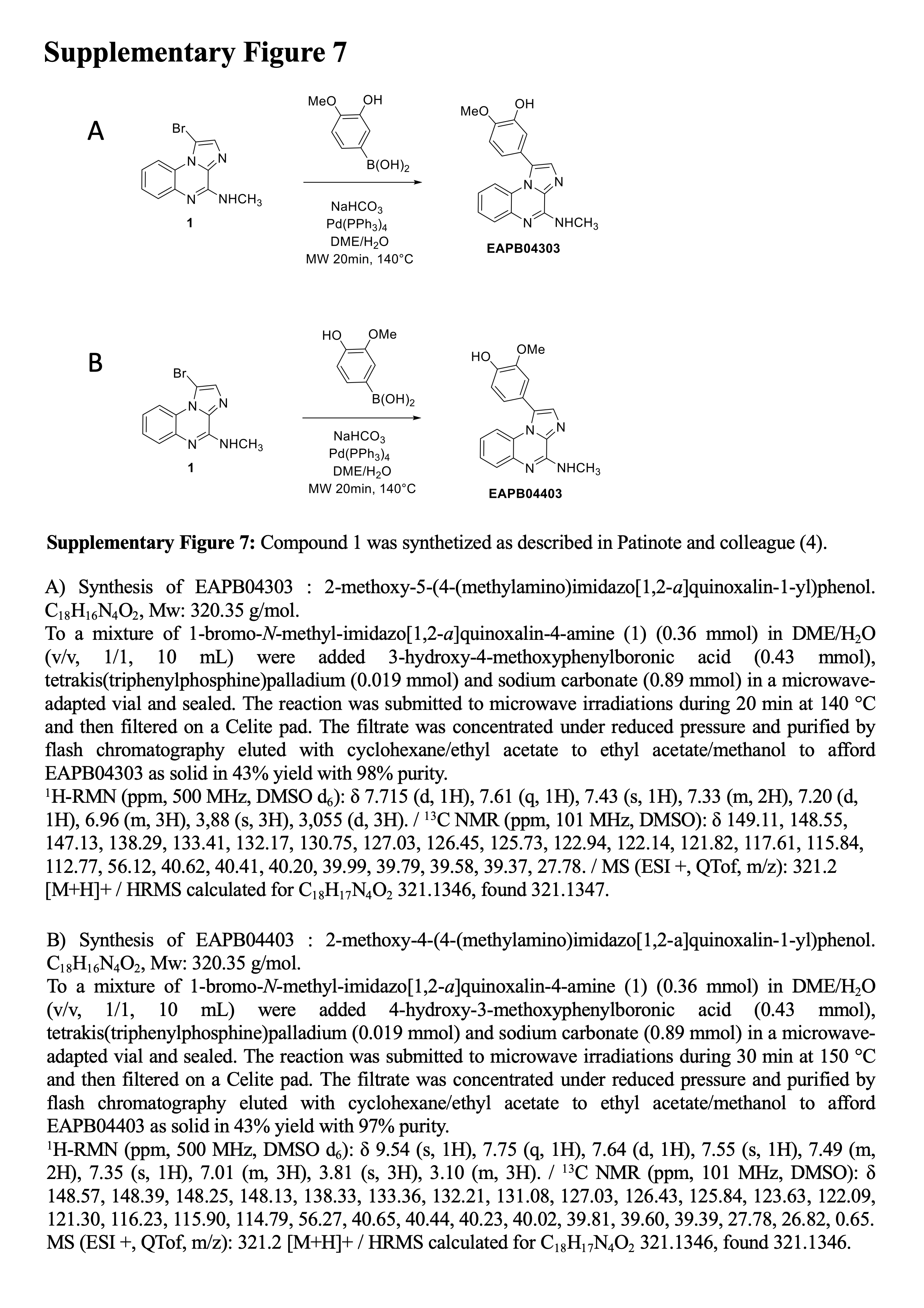

Supplement: Supplementary file 8 — supplementary Figure 7 [file 41419_2025_7747_MOESM8_ESM.tif]

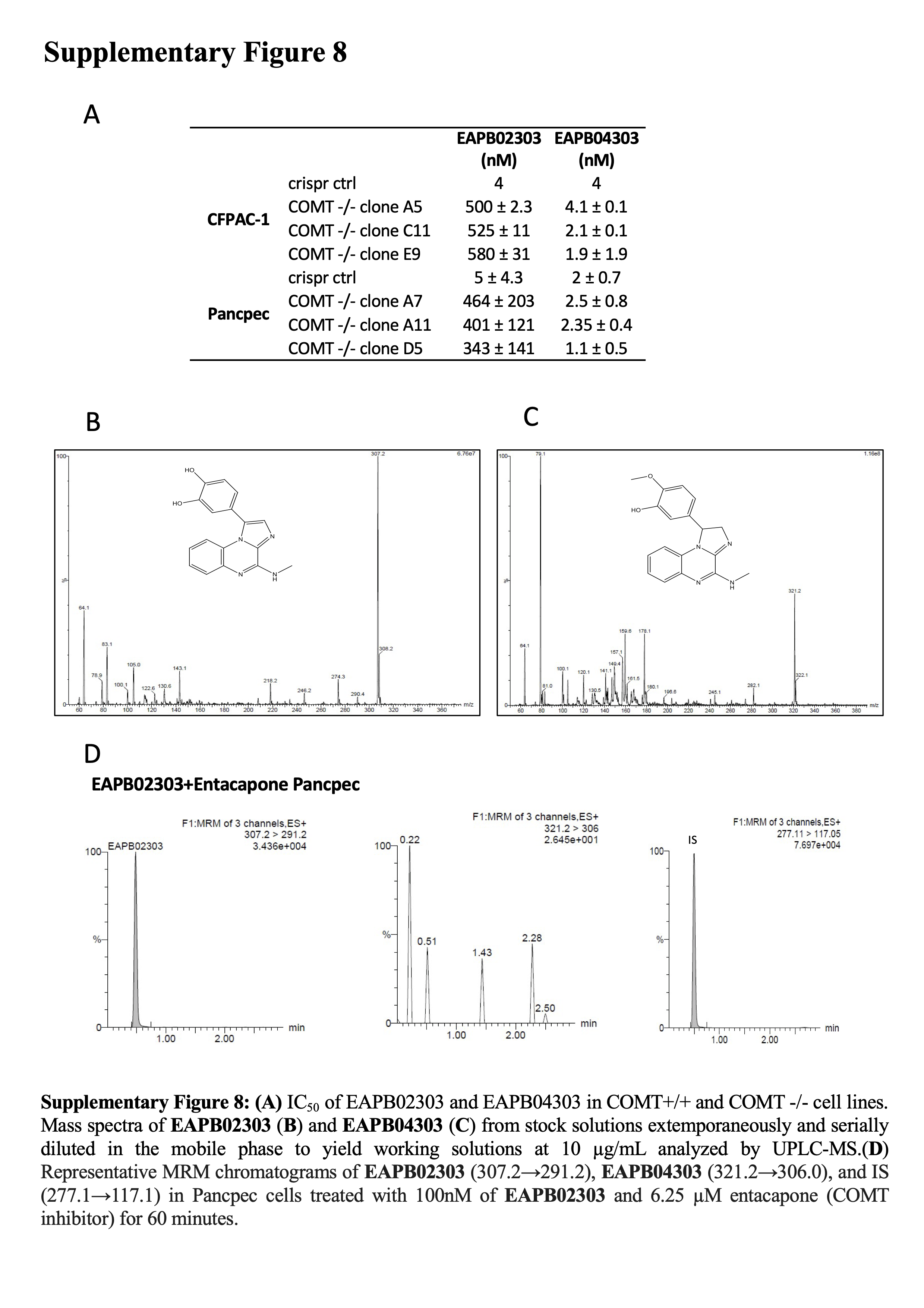

Supplement: Supplementary file 9 — supplementary Figure 8 [file 41419_2025_7747_MOESM9_ESM.tif]

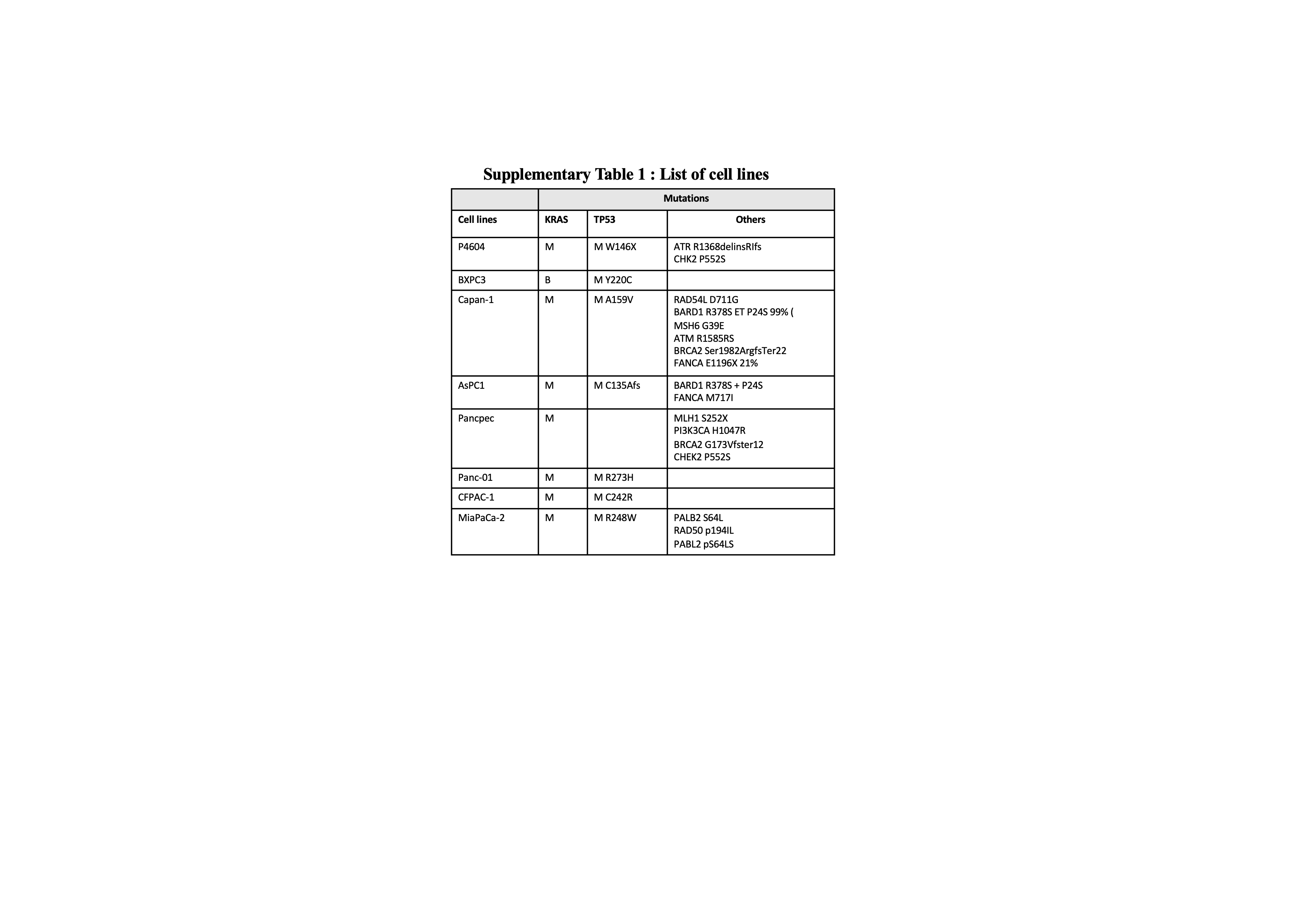

Supplement: Supplementary file 10 — supplementary Table 1 [file 41419_2025_7747_MOESM10_ESM.tif]

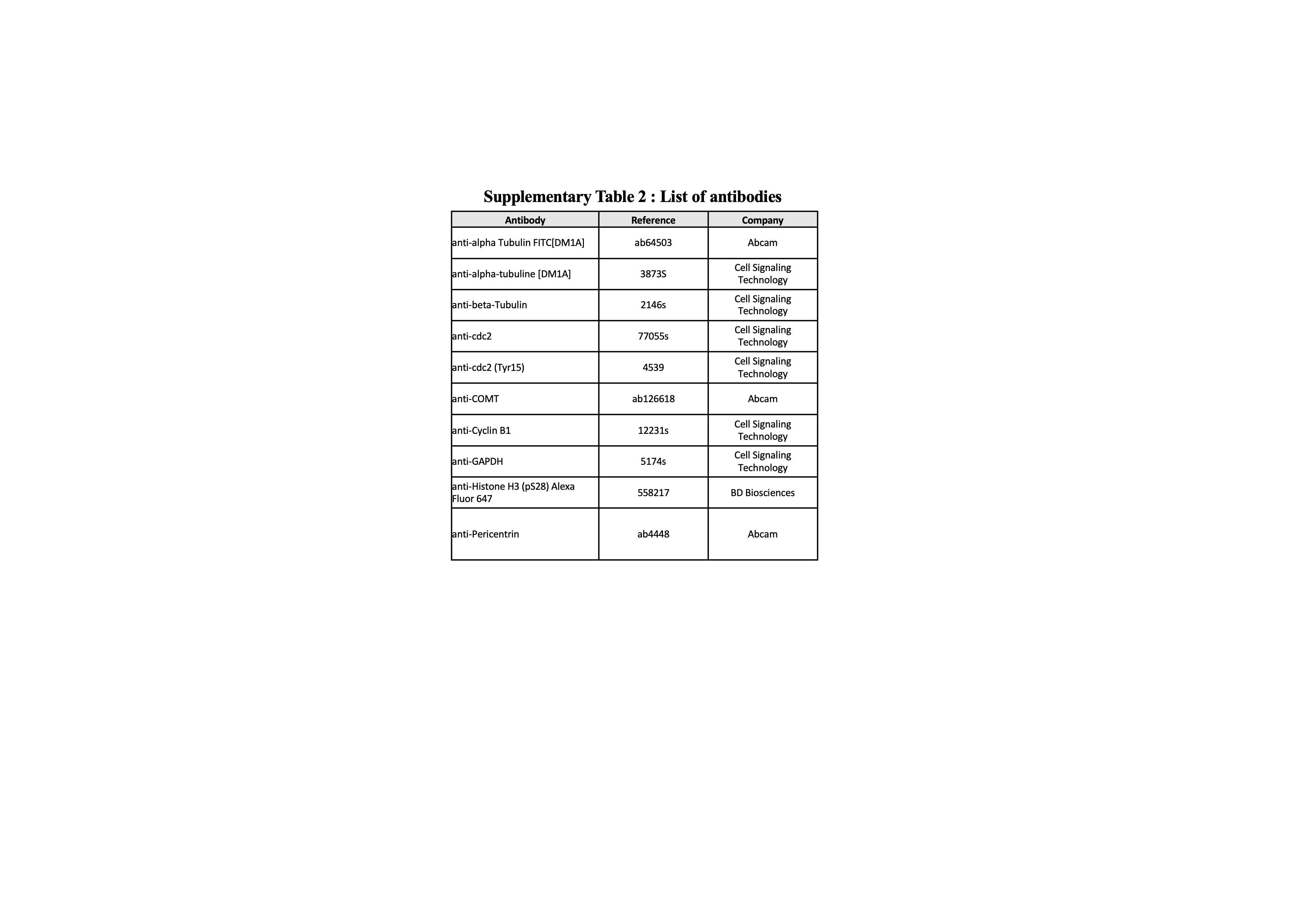

Supplement: Supplementary file 11 — supplementary Table 2 [file 41419_2025_7747_MOESM11_ESM.tif]
