# Supplementary material for: Inhibiting microtubule polymerization with EAPB02303, a prodrug activated by catechol-O-methyl transferase, enhances paclitaxel effect in pancreatic cancer models
Source: Cell Death Dis. 2025 Jun 9;16(1):441. doi: 10.1038/s41419-025-07747-1 (PMC12149313; doi:10.1038/s41419-025-07747-1)
Supplement: Supplementary file 12 — Western blots [file 41419_2025_7747_MOESM12_ESM.pdf]

- Figure 4 : cyclines

CycB1

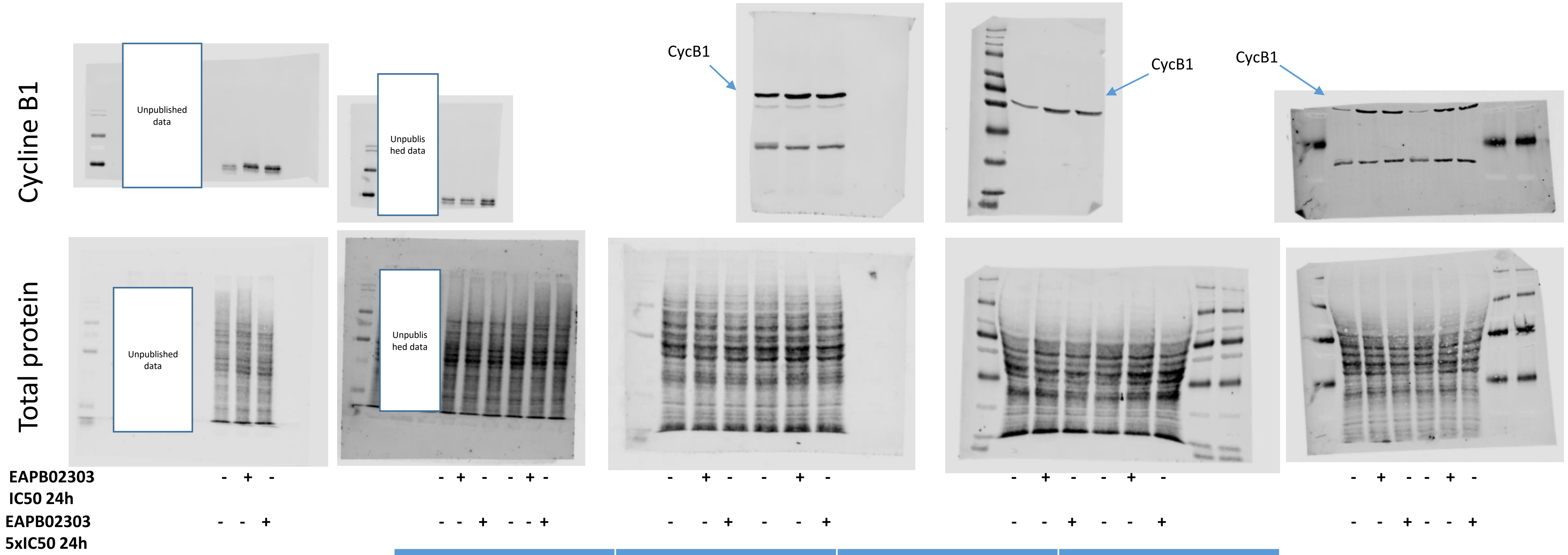

Cyclin B1	Untreated	EAPB02303 IC50 24h	EAPB02303 5*IC50 24h
n1	1	1,8	2,3
n2	1	1,03	2
n3	1	1,74	1,8
n4	1	1,8	1,8
N5 (mean of both technic replicates)	1	3,8	4,5

cdk1 total

cdk1 total

Total protein

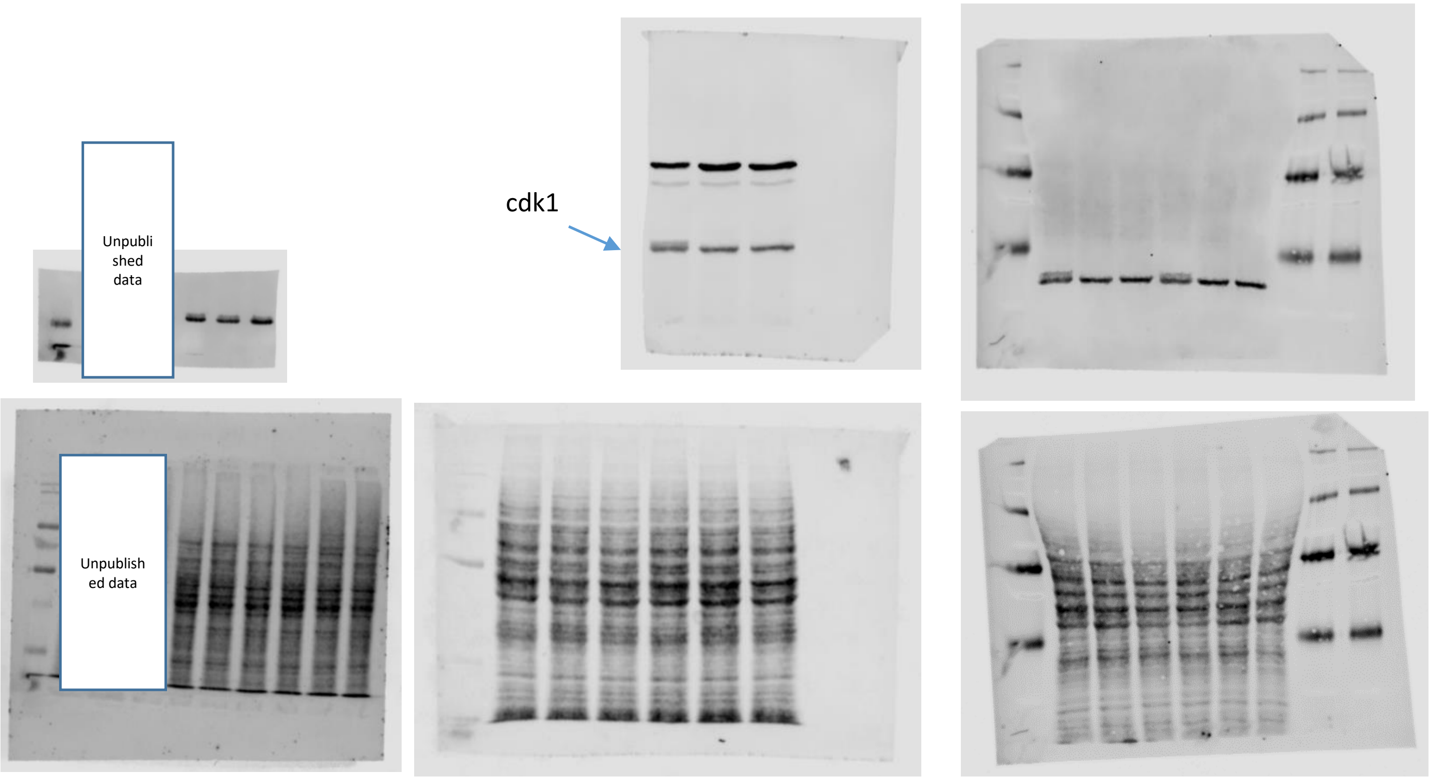

EAPB02303 IC50 24h - + -   - + -   - + - - + -

EAPB02303 5xIC50 24h - - +   - - +   - - + - - +

cdk1 total	Untreated	EAPB02303 IC50 24h	EAPB02303 5*IC50 24h
n1	1	0,9	1,2
n2	1	0,8	0,9
n3 (mean of both technic replicates)	1	0,8	1

Cdk1 15p

Cdk1 15p

Total protein

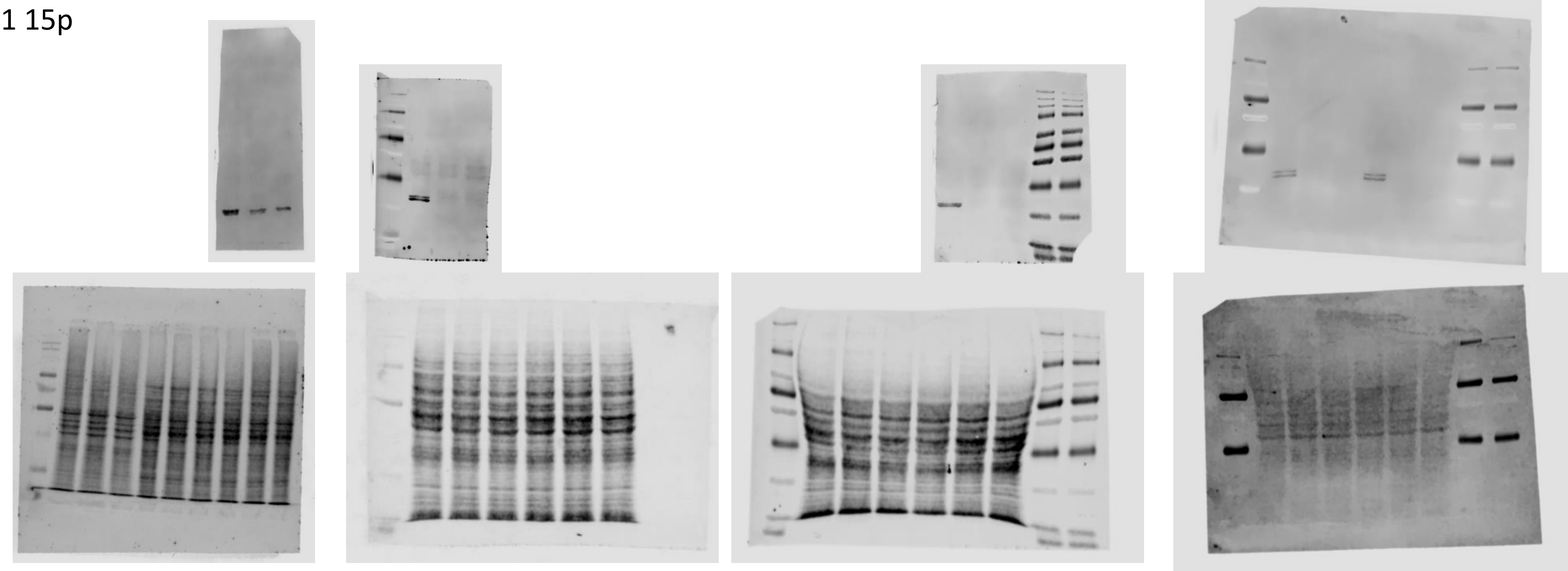

EAPB02303 IC50 24h	- + -	- + -	- + -	- + - - + -
EAPB02303 5xIC50 24h	- - +	- - +	- - +	- - + - - +

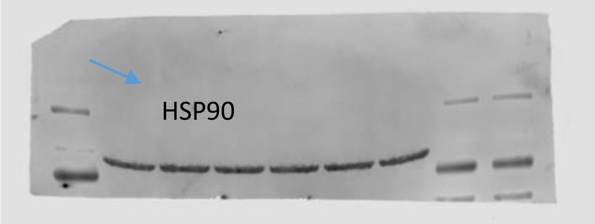

Used as supplemental loading control in regard to total protein stain low signal

cdk1-15p	Untreated	EAPB02303 IC50 24h	EAPB02303 5*IC50 24h
n1	1	0,4	0,4
n2	1	0,07	0,04
n3	1	0,06	0,08
n4 (mean of both technic replicates)	1	0,05	0,05

- Figure 4 Cellular Thermal shift assay

CETSA TUBB

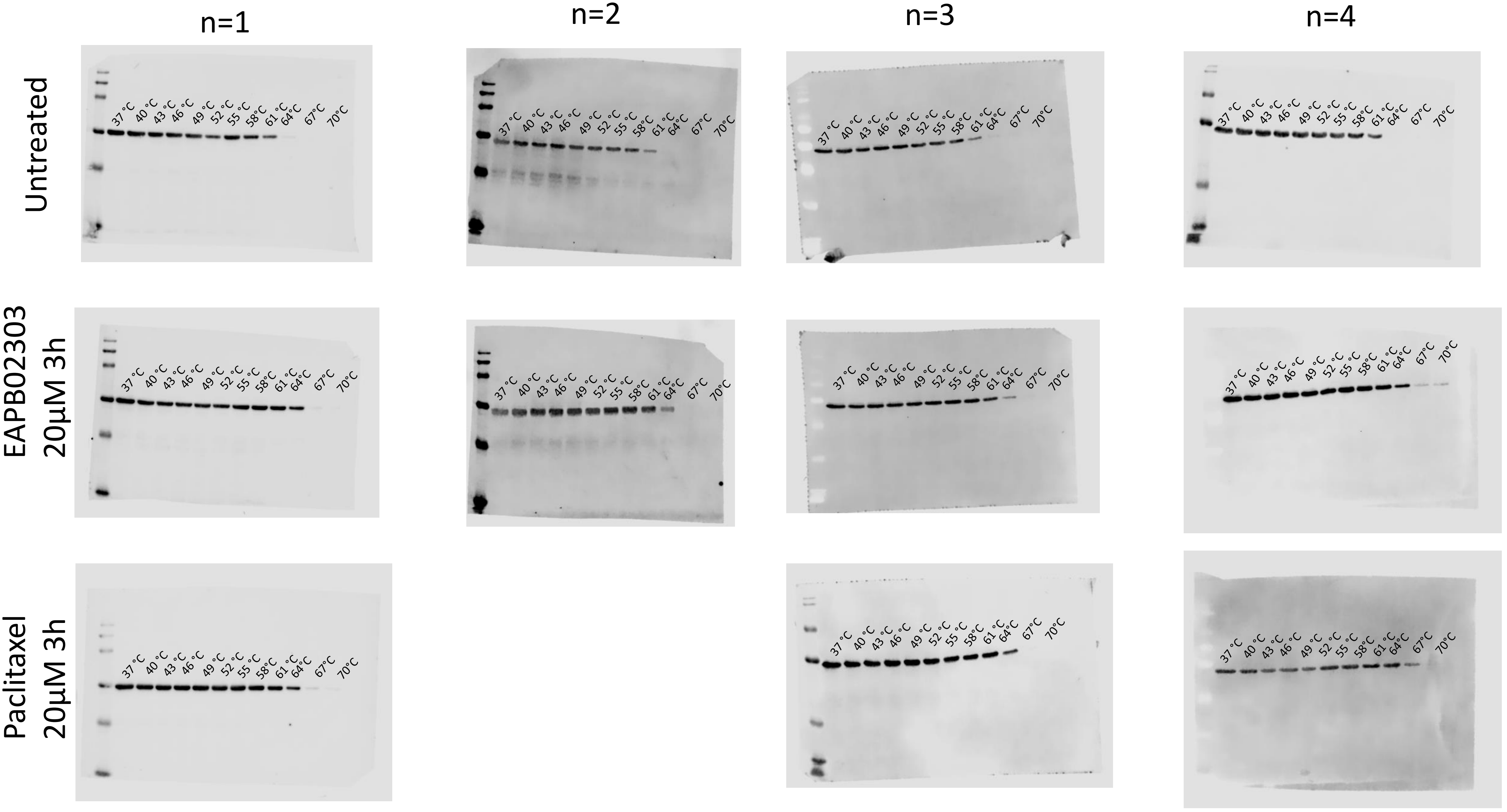

n=1
n=2
n=3
n=4
Untreated
EAPB02303 20µM 3h
Paclitaxel 20µM 3h
37 °C 40 °C 43 °C 46 °C 49 °C 52 °C 55 °C 58°C 61 °C 64°C 67°C 70°C

CETSA TUBB – Quantifications

	n1			n2			n3			n4		
37°C	NT	EAPB	Pacli	NT	EAPB	Pacli	NT	EAPB	Pacli	NT	EAPB	Pacli
40°C	119,8	129,5	105,0	82,7	86,9		119,0	116,1	116,8	105,3	111,5	127,9
43°C	97,3	90,5	94,8	108,5	97,9		103,2	82,6	99,7	98,0	99,5	101,8
46°C	82,8	80,0	100,2	108,9	115,2		77,8	101,3	83,5	96,8	88,9	70,3
49°C	72,8	71,9	102,8	107,6	116,9		106,9	85,4	102,8	93,6	87,6	84,6
52°C	63,5	68,6	102,6	84,7	110,6		101,4	83,4	114,1	87,2	91,3	67,4
55°C	43,8	67,1	86,8	83,9	105,4		79,2	92,6	90,1	72,6	77,4	134,8
58°C	76,0	95,7	89,6	84,3	120,3		74,5	98,9	76,1	58,7	78,3	112,9
61°C	55,9	109,0	89,8	71,2	118,9		66,2	86,2	80,0	70,5	86,6	131,9
64°C	33,3	93,3	75,7	27,3	90,4		27,8	53,1	83,5	41,9	71,2	121,9
67°C	1,9	60,5	42,3	0,0	32,8		0,0	16,0	35,0	0,0	39,2	39,6
70°C	0,0	1,5	2,8	0,0	0,0		0,0	0,0	0,0	0,0	4,2	0,0
	0,0	0,0	0,0	0,0	0,0		0,0	0,0	0,0	0,0	4,5	0,0

Expressed as the percentage of folded protein normalized uppon the three first temperatures of the T° range

CETSA TUBB – Statistical testing

EC50 T°

EC50 has been estimated manually by drawing the graphs, fitting linear regressin model and reporting the T° at 50% folded protein

EC50 (T°)	NT	PACLI	EAPB
	58,5	63,8	64,2
	59,3		63,1
	58,7	63	61,2
	59,5	63,7	63

Statistical testing :

Normal distrubution		
Untreatd	shapiro.test(c(58.5,59.3,58.7,59.5))	**p=0.4877**
Paclitaxel	shapiro.test(c(63.8,63,63.7))	**p=0.2196**
EAPB02303	shapiro.test(c(64.2,63.1,61.2,63))	**p=0,6152**
Variance		
Untreatd vs Paclitaxel	var.test(c(58.5,59.3,58.7,59.5),c(63.8,63,63.7))	p=0,974
Untreated vs EAPB02303	var.test(c(58.5,59.3,58.7,59.5),c(64.2,63.1,61.2,63))	p=0,1496
Student		
Untreated vs EAPB02303	t.test(c(58.5,59.3,58.7,59.5),c(64.2,63.1,61.2,63),var.equal=TRUE,alternative="two.sided")	p = 0.001124315602624.
Untreated vs Paclitaxel	t.test(c(58.5,59.3,58.7,59.5),c(63.8,63,63.7),var.equal=TRUE,alternative="two.sided")	p = 7.5132863729267E-5

- Figure 5 COMT$^{-/-}$ models

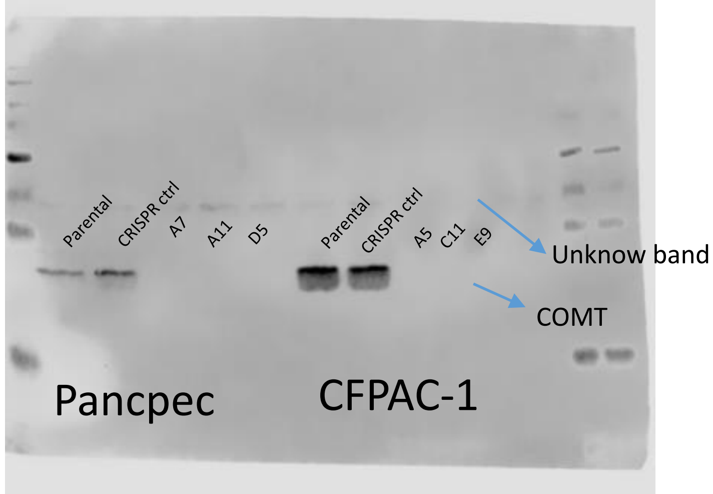

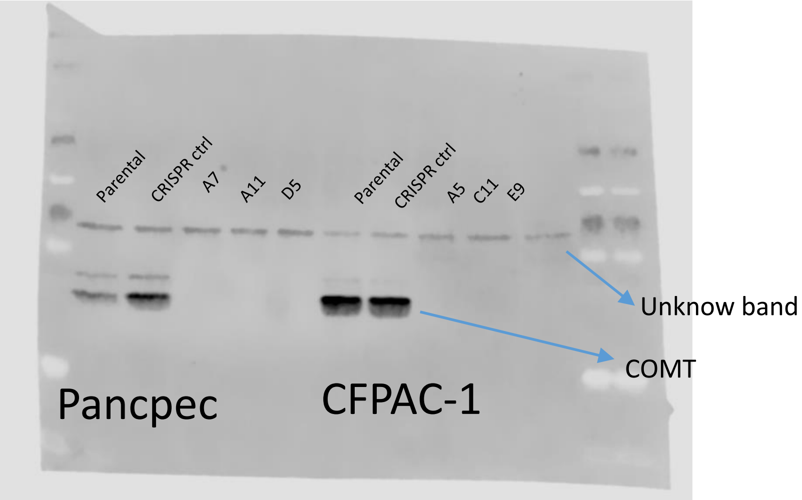

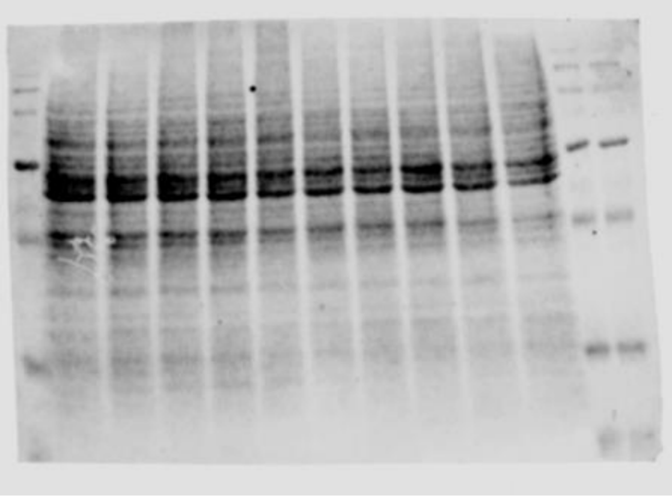

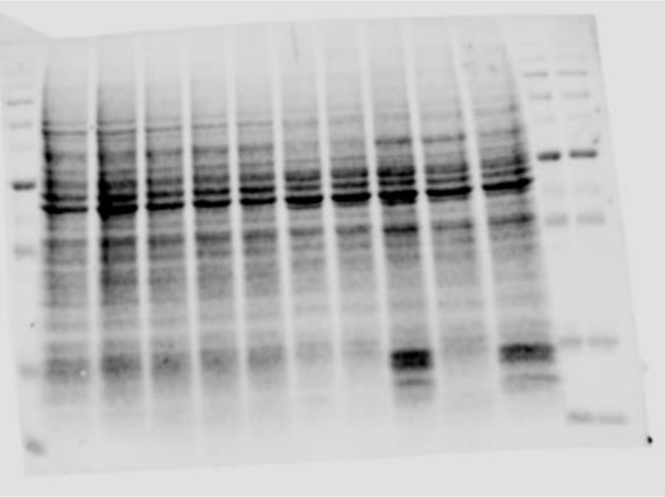

- Figure 5 ITDR Cellular Thermal Shift Assay

ITDR CETSA TUBB on CFPAC-1 COMT $^{-/-}$ (clone E9)

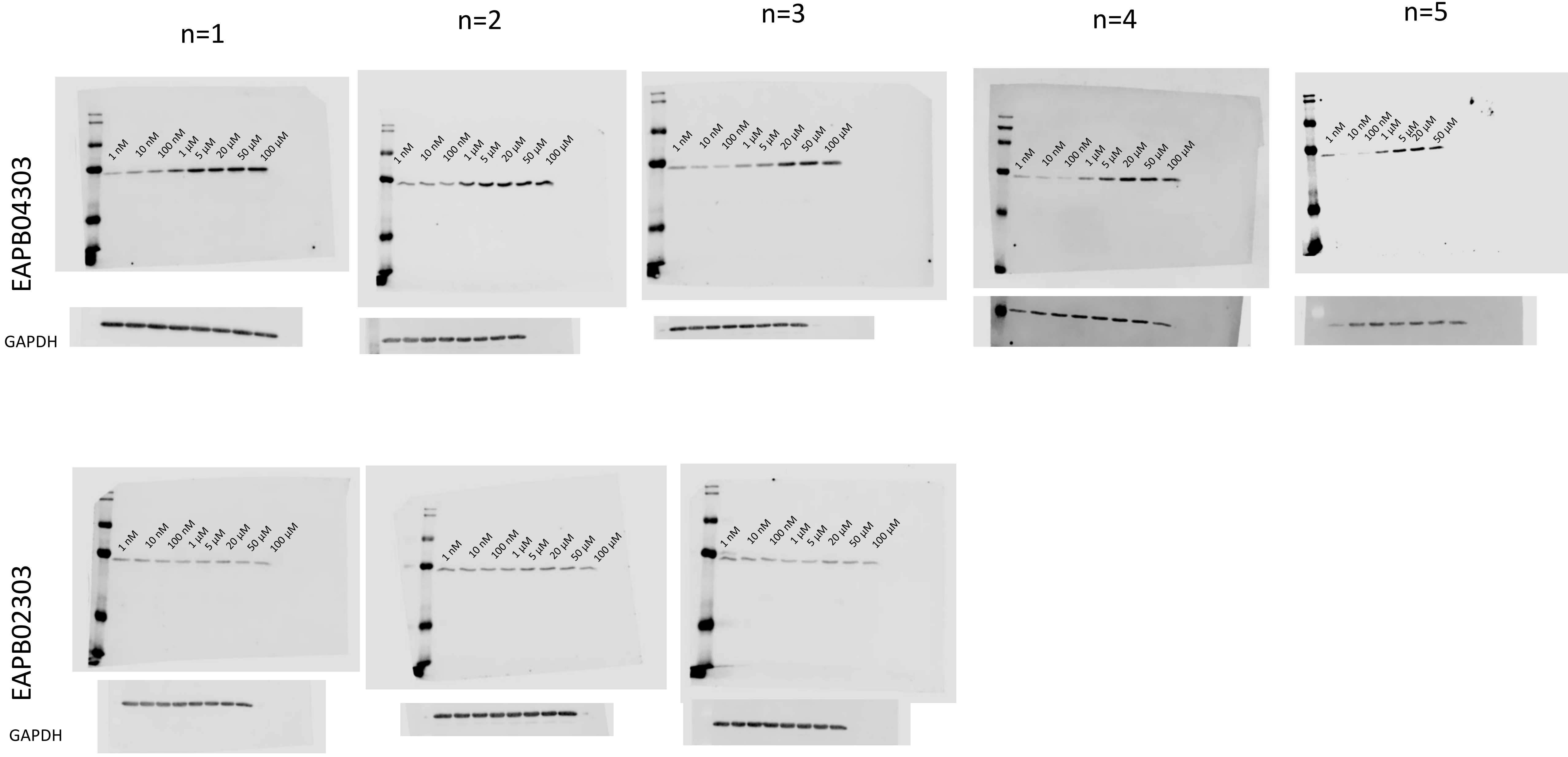

ITDR CETSA TUBB – Quantification and EC50 determination for EAPB04303

**EAPB04303**	**Signal**				
	n1	*n2*	*n3*	*n4*	*n5*
**1 nM**	11,2	45,0	23,8	removed	removed
**10 nM**	16,7	40,2	16,9	7,5	10,3
**100 nM**	18,5	23,5	11,7	1,6	11,2
**1 µM**	37,3	63,9	27,9	21,6	59,4
**5µM**	85,0	85,7	32,5	58,8	97,8
**20 µM**	86,9	102,2	106,2	123,4	116,9
**50 µM**	92,9	99,0	112,8	100,7	83,1
**100 µM**	107,1	101,0	87,2	99,3	NA
EC50	*1,9*	*1,5*	*5,7*	*5,0*	*1,8*

All values normalized to GAPDH intensity and expressed as percentage of the mean of the two highest concentrations (plateau)
EC50 were determined graphically after plotting of the percentage as a function of concentration

EC50 = 3 ± 2 µM

ITDR CETSA TUBB – Quantification and EC50 determination for EAPB04303

EAPB02303	signal		
	n1	*n2*	*n3*
**1 nM**	17,8	30,7	16,5
**10 nM**	16,7	26,2	12,8
**100 nM**	11,9	23,4	8,6
**1 µM**	9,5	23,5	6,1
**5µM**	10,8	30,8	6,6
**20 µM**	14,4	30,1	13,0
**50 µM**	9,7	25,1	10,5
**100 µM**	10,7	15,5	8,8
EC50	*NA*		

All values normalized to GAPDH intensity and expressed as percentage of the mean of the two highest concentrations (plateau)
EC50 were determined graphically after plotting of the percentage as a function of concentration

EC50 impossible to fit
